# Two RND proteins involved in heavy metal efflux in *Caulobacter crescentus* belong to separate clusters within proteobacteria

**DOI:** 10.1186/1471-2180-13-79

**Published:** 2013-04-11

**Authors:** Estela Y Valencia, Vânia S Braz, Cristiane Guzzo, Marilis V Marques

**Affiliations:** 1 Departamento de Microbiologia, Instituto de Ciências Biomédicas, Universidade de São Paulo, Av. Prof. Lineu Prestes 1374, São Paulo, SP, 05508-900, Brazil

**Keywords:** Heavy metal efflux, RND systems, *Caulobacter crescentus*, Gene expression

## Abstract

**Background:**

Heavy metal Resistance-Nodulation-Division (HME-RND) efflux systems help Gram-negative bacteria to keep the intracellular homeostasis under high metal concentrations. These proteins constitute the cytoplasmic membrane channel of the tripartite RND transport systems. *Caulobacter crescentus* NA1000 possess two HME-RND proteins, and the aim of this work was to determine their involvement in the response to cadmium, zinc, cobalt and nickel, and to analyze the phylogenetic distribution and characteristic signatures of orthologs of these two proteins.

**Results:**

Expression assays of the *czrCBA* operon showed significant induction in the presence of cadmium and zinc, and moderate induction by cobalt and nickel. The *nczCBA* operon is highly induced in the presence of nickel and cobalt, moderately induced by zinc and not induced by cadmium. Analysis of the resistance phenotype of mutant strains showed that the Δ*czrA* strain is highly sensitive to cadmium, zinc and cobalt, but resistant to nickel. The Δ*nczA* strain and the double mutant strain showed reduced growth in the presence of all metals tested. Phylogenetic analysis of the *C. crescentus* HME-RND proteins showed that CzrA-like proteins, in contrast to those similar to NczA, are almost exclusively found in the Alphaproteobacteria group, and the characteristic protein signatures of each group were highlighted.

**Conclusions:**

The *czrCBA* efflux system is involved mainly in response to cadmium and zinc with a secondary role in response to cobalt. The *nczCBA* efflux system is involved mainly in response to nickel and cobalt, with a secondary role in response to cadmium and zinc. CzrA belongs to the HME2 subfamily, which is almost exclusively found in the Alphaproteobacteria group, as shown by phylogenetic analysis. NczA belongs to the HME1 subfamily which is more widespread among diverse Proteobacteria groups. Each of these subfamilies present distinctive amino acid signatures.

## Background

From a physiological point of view, metals fall into three main categories, namely essential and non-toxic (e.g. Ca^2+^ and Mg^2+^); essential, but harmful at high concentrations (e.g. Fe^2+^, Mn^2+^, Zn^2+^, Cu^2+^, Co^2+^, Ni^2+^ and Mo^2+^), and toxic (e.g. Hg^2+^ or Cd^2+^) [1]. However, at high concentrations, both essential and nonessential metals can be harmful to the cell, damaging the cell membrane, the structure of DNA, or changing the specificity of enzymes [2-4]. The microorganisms have developed homeostasis systems in order to maintain an optimal intracellular concentration of metals. This is achieved through controlling the processes of transport, intracellular trafficking, efflux and conservation, ensuring its bioavailability to cellular processes and preventing damage to cellular components [5].

Studies support a role for horizontal gene transfer (HGT) in the evolution of metal homeostasis in Proteobacteria, along with the identification of putative genomic islands (GIs), with examples in *Cupriavidus metallidurans*, *Pseudomonas putida* KT2440 and *Comamonas testosteroni* S44 [6-9]. In fact, many microorganisms have genes located on chromosomes, plasmids, or transposons encoding specific traits conferring resistance to a variety of metal ions [3].

Efflux is one of the main approaches used by bacteria to control internal metal ion concentrations, and several efflux systems have been described in bacteria. The P-type ATPases use ATP hydrolysis to promote ion transport and have been identified in efflux of both mono- and divalent cations from the cytoplasm [10-13]. The Cation Diffusion Facilitator (CDF) are chemiosmotic ion/proton exchangers that present six transmembrane helices and are involved in the efflux of divalent metal cations [11,14,15].

In Gram-negative bacteria, the Resistance-Nodulation-Division superfamily (RND) includes systems that confer resistance to antibiotics and metals, and it is composed of a tripartite protein complex: an RND protein, located in the cytoplasmic membrane, a periplasmic membrane fusion protein (MFP) and an outer-membrane channel protein (OMP) [16-18]. These components form a channel that spans both membranes and the periplasmic space [18-21]. Based on the nature of their substrate, the RND superfamily was divided into seven families, of which the hydrophobe/amphiphile efflux (HAE), and the heavy-metal efflux (HME) have been extensively studied [17,22]. Nies further subdivided the HME-RND proteins into sub-groups, according to the substrate they transport: HME1 (Zn^2+^, Co^2+^, Cd^2+^), HME2 (Co^2+^, Ni^2+^), HME3a (divalent cations), HME3b (monovalent cations), HME4 (Cu^+^ ou Ag^+^) and HME5 (Ni^2+^) [14].

The cytoplasmic membrane RND proteins have 12 transmembrane alpha helices (TMH), among which TMH IV contains amino acid residues that are conserved in most RND proteins [17]. The HME1-RND and HME2-RND have the same motifs, DFG-DGA-VEN, present in proteins CzcA (HME1) or CnrA and NccA (HME2) [14,23]. Both aspartate residues and the glutamate residue in TMH IV of CzcA are required for proton/substrate-antiport, suggesting that they are probably involved in proton translocation [14,23,24]. A model for cation transport by an HME-RND was recently proposed for the copper transporter CusA, in which the metal ion moves along a pathway of methionine residues, causing significant conformational changes in both the periplasmic and transmembrane domains [25]. These systems are proposed to promote the efflux of both cytoplasmic and periplasmic substrates, transporting of the substrate either via the RND protein or in some cases via the membrane fusion protein with the aid of periplasmic metal chaperones [14,24].

The best characterized RND heavy metal efflux systems are mainly those from *Cupriavidus* (previously called *Ralstonia* and *Alcaligenes)*: CzcCBA (Cd^2+^, Zn^2+^, and Co^2+^ resistance) from *Ralstonia metallidurans* CH34 [26-28]; CnrCBA (Ni^2+^ and Co^2+^) from *Ralstonia eutropha*[29,30]; NccCBA (Ni^2+^, Co^2+^ and Cd^2+^) from *Alcaligenes xylosoxidans* 31A [31]. However, other systems such as *Pseudomonas aeruginosa* Czr (Cd^2+^ and Zn^2+^ resistance) [32]; and *Helicobacter pylori* Czn (Cd^2+^, Zn^2+^ and Ni^2+^ resistance) were also studied [33].

In order to better understand the role of the RND efflux systems in the export of divalent cations in other Proteobacteria, we investigated the role of two HME-RND systems present in the Alphaproteobacterium *Caulobacter crescentus.* A previous bioinformatics analysis made by Nies (2003) through comparison of the genomes of 63 prokaryotes (Archaea and Bacteria) with the genome of *C. metallidurans,* identified seven ORFs encoding putative RND proteins in *C. crescentus* CB15 of which two, CC2724 (corresponding to CCNA_02809 in the derivative strain NA1000; here called CzrA) and CC2390 (CCNA_02473; here called NczA), belong to the HME subgroup. Previous works from our group [34] identified that the *czrCBA* locus is involved in resistance to cadmium and zinc and is induced by these cations, and other reports [35] confirmed that this operon is induced by cadmium.

In this work, we have characterized both of these systems by constructing null mutant strains of the respective RND-encoding genes and evaluating their resistance to cadmium, zinc, cobalt and nickel. We have also studied the pattern of gene expression of both operons in response to each metal. The results showed that the two proteins have different responses to metals both in resistance and in expression, suggesting distinct but somewhat overlapping roles for each protein. Moreover, a phylogenetic analysis showed that these proteins belong to two distinct clusters, and that each group presents distinctive amino acid signatures.

## Results and discussion

### Comparative analysis of *czr* and *ncz* clusters

**Figure 1:**
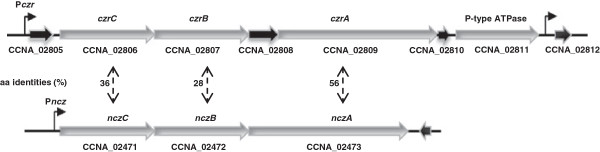
**Schematic representation of the *czr *and *ncz *loci.** The *czr* locus includes six predicted ORFs (CCNA_02805 to CCNA_02811) that probably constitute a putative operon. The putative promoter regions are indicated by bent arrows, upstream of CCNA_02805 (P*czr*), CCNA_02806 (Pczr*), and CCNA_02812. The *ncz* locus includes a putative operon containing three ORFs (CCNA_02471 to CCNA_02473), transcribed from the P*ncz* promoter. The percentages of amino acid identity between each paralog are indicated by two-way arrows.

In an extensive analysis of putative heavy-metal exporters in microbial genomes, Nies [14] performed a BLAST search against the CzcA from *R. metallidurans*, confirming with multiple alignments and checking for the presence of specific signatures, to assign proteins into the RND family. This global search identified seven RND proteins in the genome of *C. crescentus* but only the proteins encoded by the CCNA_02809 gene (*czrA*) and the CCNA_02473 gene (*nczA)* contained the conserved motifs DFG-GAD-VEN, belonging to subgroup HME-RND [14]. As shown in Figure 1, these genes belong to two putative operons containing the *czcCBA*-related genes. In both *czcCBA-*related operons analyzed, no regulatory genes are found in the vicinity, in contrast to what was described for the *cnr* operon of *R.metallidurans* CH34 (*cnrYXHCBA*), *czc* of *R. metallidurans* and *A. eutrophus* (*czcNICBADR*S and *czcCBADRS*) and *ncc* of *A. xilosoxidans* 31A (*nccYXHCBAN*) [27,30,31,36].

Amino acid alignments showed that these paralogous share very low overall identity: CCNA_02806 and CCNA_02471 (CzrC and NczC, outer membrane factor), 36% identity; CCNA_02807 and CCNA_02472 (CzrB and NczB, membrane fusion protein), 28% identity; and CCNA_02809 and CCNA_02473 (CzrA and NczA, RND protein) 56% identity. Moreover, the *czr* locus contains three additional genes encoding putative hypothetical proteins (CCNA_02805, CCNA_02808 and CCNA_02810). Orhtologues of CCNA_02805 are found in this locus in *Phenylobacterium zucineum* and in *Stenotrophomonas maltophilia*, but no orthologs of CCNA_02808 are found in this locus outside of the Caulobacteraceae. The CCNA_02810 is a putative ATP-binding conserved protein that possesses a domain of unknown function. The low similarity among proteins encoded in these two loci suggests that they have diverged substantially, and that they may have acquired specialized roles in maintaining metal homeostasis.

### Identification of *czr* and *ncz* promoter regions

**Figure 2:**
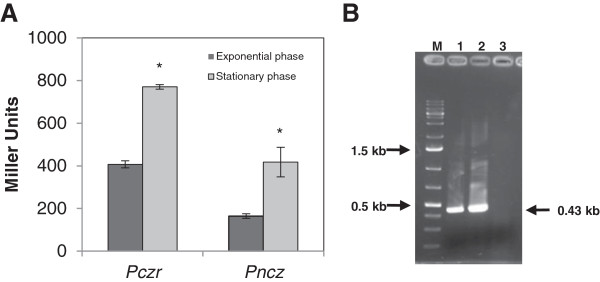
**Characterization of the *czr *and *ncz *promoter regions.** (**A**) Beta-galactosidase activity assay of transcription fusions of P*czr* and P*ncz* to the *lacZ* reporter gene. Cells were grown in PYE medium and samples were taken at midlog phase and stationary phase (24 h) for assaying β-galactosidase as described [38]. The background activity for plasmid alone is around 200 Miller Units. Asterisks indicate results significantly different between the two growth phases within each promoter fusion (p ≤ 0.05). (**B**) Determination of co-transcription of CCNA02805 and CCNA_02806 by amplification with primers RND3 and RND4. Lane 1, PCR amplification using cDNA previously synthesized with Reverse Transcriptase from total RNA from the NA1000 strain; lane 2, PCR amplification from total NA1000 genomic DNA (positive control); lane 3, PCR amplification from total RNA from the NA1000 strain (negative control). The 0.43 kb fragment corresponding to the amplified products is indicated.

The putative ORF CCNA_02805 is separated from *czrC* by 57 bp, which raised questions as to the location of the promoter region for the *czrCBA* operon. In order to identify the *czrCBA* and *nczCBA* promoter regions and perform gene expression analysis, transcriptional fusions to the *lacZ* reporter gene in the pRK*lacZ290* vector were constructed. The fusions were constructed as folows: P*nczC* (containing the region upstream of *nczC*); P*czr* (containing the region upstream of CCNA_02805) (Figure 1); and Pczr* (containing the region upstream of *czrC*). *C. crescentus* NA1000 carrying each transcriptional fusion were used in β-galactosidase activity assays (Figure 2A). The results showed that P*nczC/lacZ* fusion generated β-galactosidase activities of 164 and 418 Miller units at exponential and stationary phase, respectively. P*czr/lacZ* fusion generated β-galactosidase activities of 407 (exponential phase) and 770 (stationary phase) Miller units; however, the Pczr*/lacZ construct generated only the same activity as the vector alone (data not shown). The results indicate that the intergenic region between CCNA_02805 and *czrC* genes lacks a promoter, and the *czrCBA* operon expression is driven by a promoter upstream of CCNA_02805. In fact, a global analysis in search for *C. crescentus* metal-inducible promoters identified transcription start sites upstream of CCNA_02805 and CCNA_02812, but none were detected upstream of *czrA, czrB* or *czrC*[37]. Moreover, transcription from both these sites increased upon cadmium treatment, and a putative sequence motif (m_7) was identified in the region upstream of CCNA_02805 that is conserved upstream of other cadmium-induced genes [37].

To confirm that CCNA_02805 belongs to the *czrCBA* operon, an RT-PCR analysis was carried out using primers within the predicted coding regions of CCNA_02805 and *czrC* (Figure 2B). The results confirmed that there is a transcript encompassing CCNA_02805 and *czrC*. Since there are no gaps between *czrC*-*czrB* and *czrB*-*czrA* (the same goes for *nczCBA)*, we conclude that at least these genes belong to putative operons. We cannot exclude the possibility that CCNA_02811 (encoding a putative Cd^2+^/Zn^2+^-exporting P-type ATPase) is co-transcribed with *czrCBA*, although the distance between CCNA_02810 and CCNA_02811 is 63 bp. These results agree with the results reported previously that transposon insertions into either CCNA_02805, CCNA_02807 or CCNA_02809 caused a similar phenotype of increased sensitivity to cadmium [34].

### Determination of gene expression in response to metals

**Figure 3:**
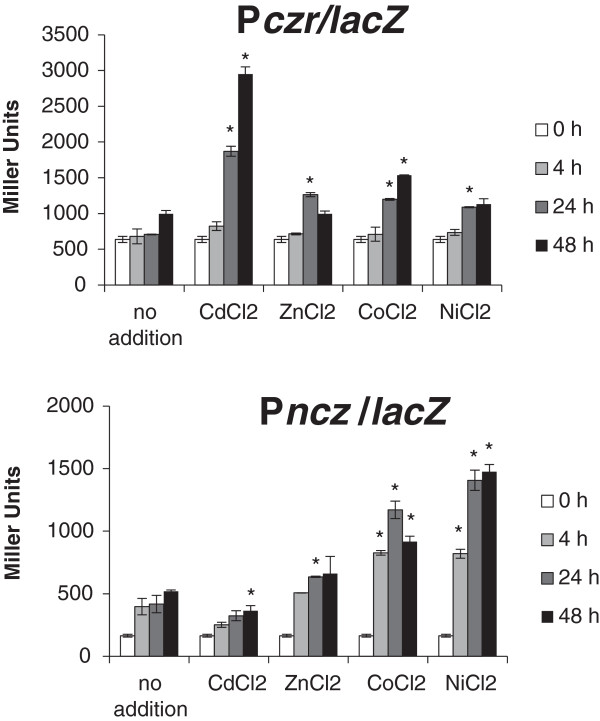
**Induction of gene expression by divalent cations.** The reporter *lacZ* gene expression driven by promoters P*czr* and P*ncz* was evaluated by β-galactosidase activity assays in the presence of different divalent cations. The results shown are the average of at least three experiments. Error bars indicate standard deviations. Metal concentrations were: CdCl_2_, 10 μM; ZnCl_2_, 100 μM; CoCl_2_, 100 μM; NiCl_2_, 100 μM. Asterisks indicate results significantly different than those of of the same time points without metal (p ≤ 0.05).

To determine whether expression driven by P*czr* and P*ncz* varied in response to different divalent cations, cultures of *C. crescentus* NA1000 harboring each transcriptional fusion were grown in PYE medium up to an OD_600_ = 0.5, and were divided into equal aliquots. Each aliquot was then added of the corresponding metal (final concentrations of 10 μM CdCl_2_, 100 μM ZnCl_2_, 100 μM CoCl_2_ or 100 μM NiCl_2_). β-galactosidase activity was determined at several time points after metal addition, and expression was evaluated relative to expression at the same points without metal addition (control). The results are shown in Figure 3. In the presence of CdCl_2_ the *ncz* operon was not induced at all times tested, in contrast to the *czr* operon, which is induced 2.5-fold after 24 h. In the presence of ZnCl_2_ both operons showed a small induction at the 24 h time point: *ncz* 1.5-fold, and *czr* 1.7-fold. Interestingly, in the presence of CoCl_2_ and NiCl_2_ the *ncz* operon demonstrated a rapid and greater induction at all times tested, reaching 2.8-fold (24 h with CoCl_2_) and 3-fold (24 h with NiCl_2_). Nevertheless, the *czr* operon showed modest induction at 24 h of exposure to metal (1.6-fold with CoCl_2_ and 1.5-fold with NiCl_2_).

These results suggest that these two RND efflux systems have different roles in response to metal. The *czr* operon seems to be important mainly for the response to cadmium and zinc, whereas the *ncz* operon for the response to cobalt and nickel, since it was highly and quickly induced by these metals. A whole-genome transcriptional analysis upon heavy metal stresses (chromium, cadmium, selenium, and uranium) showed that the cluster CCNA_02806-CCNA_02812 (including the *czr* operon and a gene encoding a P-type ATPase) is highly induced in response to cadmium [35]. In our previous work, β-galactosidase assays using the *lacZ* gene from the inserted transposon showed an induction of all genes by cadmium after 24 h [34]. The present work confirmed previous data for the *czr* regulation by zinc and cadmium, and further demonstrated that it is also induced by nickel and cobalt to a minor degree. This is also the first determination of the *ncz* operon induction by cobalt and nickel.

### Roles of each HME-RND system in metal resistance

**Figure 4:**
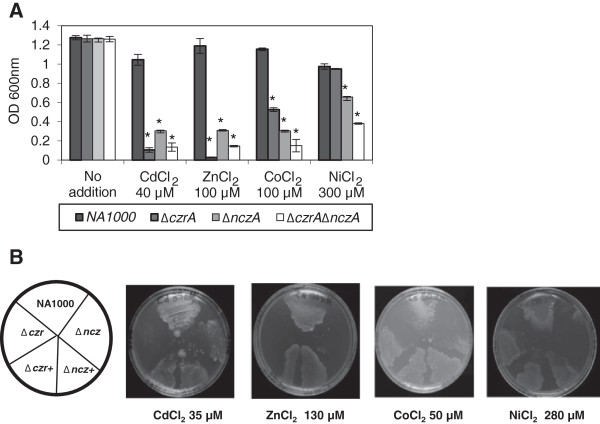
**Growth phenotype of the mutant strains.** (**A**) Cultures of *C. crescentus* strains NA1000 (wild type), Δ*czrA*, Δ*nczA*, and the double mutant Δ*czrA*Δ*nczA* at an initial OD of 0.05 were inoculated into PYE medium with or without the indicated concentrations of metal salts. The cultures were incubated at 30°C for 24 h, and then growth was assessed by determination of OD at 600 nm. The results shown are the average of two experiments. Error bars indicate standard deviations. Asterisks indicate results significantly different than those of of the same time points without metal (p ≤ 0.05). (**B**) Equal amounts of cells from cultures of *C. crescentus* strains NA1000, Δ*czrA*, Δ*nczA*, and the two complemented strains Δ*czrA +* and Δ*nczA +* were streaked on solid PYE medium. The plates were incubated at 30°C for 72 h before the pictures were taken.

In order to study the effect of metal ions on bacterial growth, the parental strain NA1000, as well as the single Δ*czrA* and Δ*nczA* and double Δ*czrA*Δ*nczA* mutant strains were grown in PYE medium with or without each individually added metal. All cultures started at the same optical density, and after 24 h growth of the strains was determined by measurement of the OD_600 nm_ (Figure 4A). In comparison to the control (without addition of metal), the NA1000 strain showed a small reduction in growth only in the presence of 40 μM CdCl_2_ (19% reduction) or 300 μM NiCl_2_ (23% reduction), being only slightly sensitive to the other metal concentrations tested. The Δ*czrA* strain showed a severe reduction in growth in the presence of 40 μM CdCl_2_ (91%) and 100 μM ZnCl_2_ (97%), exhibiting an intermediate sensitivity to 100 μM CoCl_2_ (58% reduction) and resistance to 300 μM nickel (24% reduction) comparable to the parental strain. On the other hand, Δ*nczA* had a more pronounced reduction in growth in 100 μM CoCl_2_ (76%), 40 μM CdCl_2_ (76%) and 100 μM ZnCl_2_ (75%) and showed a 48% reduction in growth with 300 μM NiCl_2_. However, it showed higher resistance to CdCl_2_ and ZnCl_2_ than the Δ*czrA* strain. As expected, the Δ*czrA*Δ*nczA* strain had growth severely affected in the presence of all metals tested.

These data, taken together with the expression profile of each operon, indicate that *czrA* is responsible mainly for cadmium and zinc efflux and has a secondary role in resistance to cobalt, whereas *nczA* is responsible mainly for nickel, and cobalt efflux with a secondary role in resistance to zinc and cadmium. To confirm the involvement of *czrA* and *nczA* in metal resistance, complementation analyses were performed for each gene. The strains harbouring the empty vector or the vector with the complementing copy of each gene were grown in PYE-kanamycin medium supplemented with 0.2% xylose and addition of the metal tested for gene induction. Figure 4B shows that complemented strains were able to grow similarly to NA1000 strain, whereas Δ*czrA* strain did not grow in CdCl_2_ and ZnCl_2_, and the Δ*nczA* strain presented reduced growth in the presence of ZnCl_2_, CoCl_2_ and NiCl_2_.

The presence of two related transport systems in the genome suggests that they would improve the capacity of *C. crescentus* to resist to high concentration of metals, agreeing with the notion that they are complementary in function.

### Characterization and distribution among proteobacteria

The CCNA_02805-02810 cluster is located at the end of a 60-kb genomic island, identified in the annotation of the corresponding strain *C. crescentus* CB15 genome [39], indicating that at least one of these *C. crescentus* RND efflux system may have been acquired by horizontal gene transfer. This confirms a common association of these genes to mobile genetic elements, as discussed for other bacteria [7,8].

**Figure 5:**
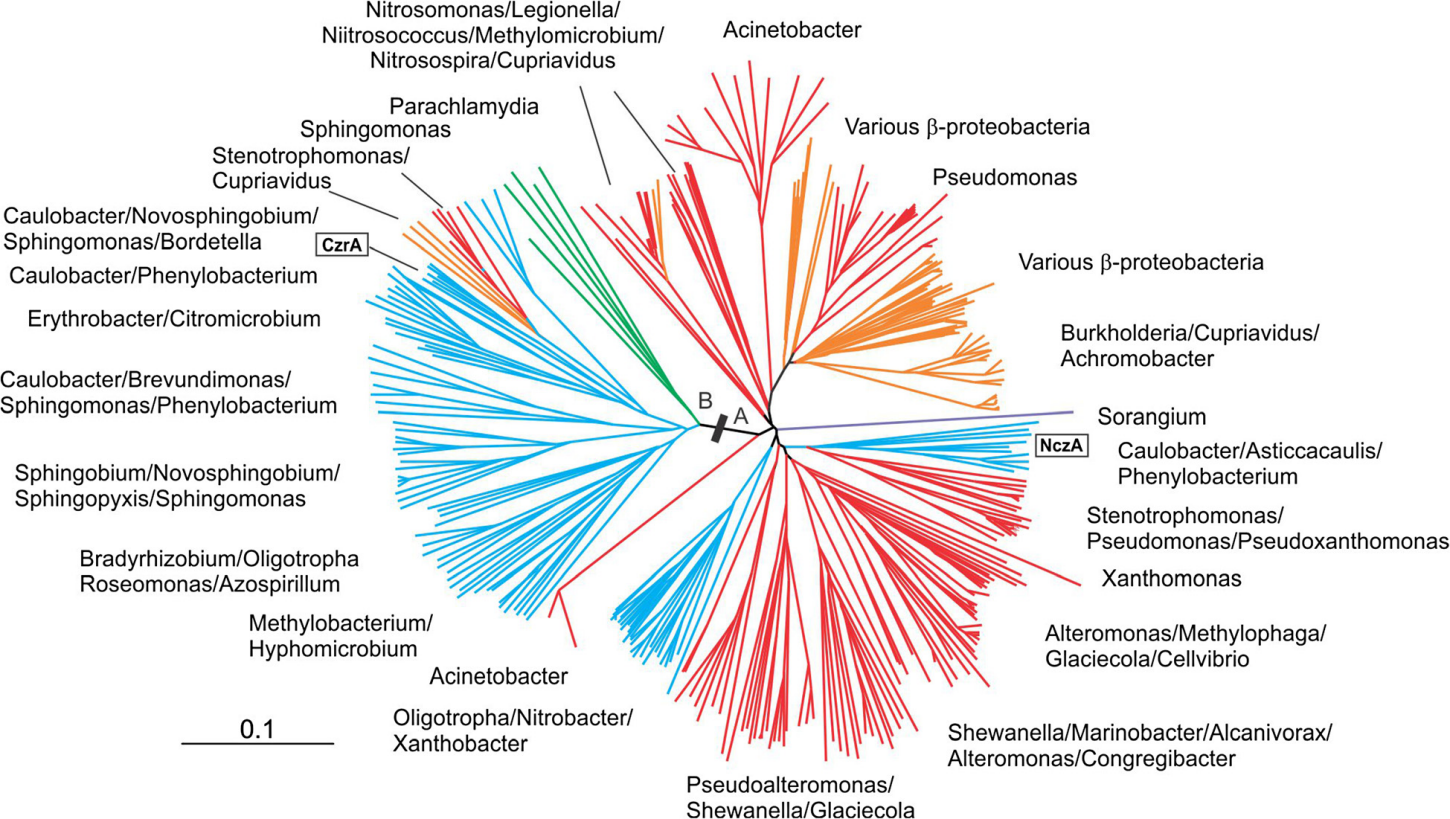
**Phylogenetic analyses of CzrA and NczA.** Phylogenetic tree for the RND proteins was constructed using a neighbor-joining method. The tree was generated from multiple sequence alignment of protein sequences with higher than 55% identity to either *C. crescentus* CzrA or NczA, and the distances were calculated using CLUSTALX [40]. The branches were color-coded as follows: blue, Alphaproteobacteria; red, Gammaproteobacteria; orange, Betaproteobacteria; green, Chlamidiales. Some of the most prevalent genera present in each branch of the tree are indicated. The two separate clusters corresponding to either *C. crescentus* orthologs are indicated as follows: **A**, NczA orthologous group; **B**, CzrA orthologous group.

To investigate the origins of these two *C. crescentus* HME-RND proteins, we performed a phylogenetic analysis of CzrA and NczA, including in the analysis sequences from orthologs with at least 55% identity to either protein. The complete list of protein sequences used can be found in Additional file 1: Table S1. This criterion was chosen given the fact that they both share this percentage of identity, but one must take into consideration that the analysis did not include all the sequences of members of the HME-RND family in the databases, although we believe that most of the protein sequences belonging to group B have been included. The analysis showed that they group into two very distinct branches, along with orthologs from other Proteobacterial groups (Figure 5). Interestingly, the two branches present a remarkable difference in the number and variety of genera included. The CzrA orthologs group in a branch (labeled B in Figure 5) composed mainly of members from the Alphaproteobacteria, and at the base of this branch are sequences from *Parachlamidia* and *Micavibrio*. On the other hand, the larger A branch is composed of sequences from much more diverse genera, including members of the Alpha, Beta and Gamma, and a single sequence from Delta-Proteobacteria. We also observed that the presence of multiple paralogs is a common trend among Alphaproteobacteria, with many genera containing representatives from both groups. Interestingly, HME-RND proteins previously identified in the *Cupriavidus* group also clustered separately, with the HME1-RND proteins in the A branch and the HME2-RND proteins emerging in a branch within the Alphaproteobacteria in the B branch. This, together with the fact that the HME2-RND genes from *Cupriavidus* and other Beta and Gamma-Proteobacteria are also found in plasmids [8], clearly indicate the acquisition of these genes by lateral transfer.

We observed no correlation between the two phylogenetic groups A and B and the response to different types of metals of the RND proteins already characterized. *C. crescentus* NczA, which is important for nickel and cobalt resistance, clustered in group A with *C. metallidurans* CH34 CzcA, which is involved in Cd^2+^/Zn^2+^/Co^2+^ resistance [26-28]. Similarly, *C. crescentus* CzrA, important for Cd^2+^/Zn^2+^ resistance, clustered in group B with CnrA from *C. metallidurans* CH34, which confers resistance to Ni^2+^ and Co^2+^, and with NccA from *A. xylosoxidans* 31A which confers Ni^2+^/Co^2+^/Cd^2+^ resistance [31,41]. It must be noticed, however, that we observed two separate branches within group A (Figure 5), which include different genera of the gamma-Proteobacteria and only one contains protein sequences from beta-Proteobacteria (such as *C. metallidurans* CzcA). We cannot exclude the possibility that these two sub-groups could show some correlation with metal specificity, but more experimental work with representative proteins from each group is necessary to clarify that.

**Figure 6:**
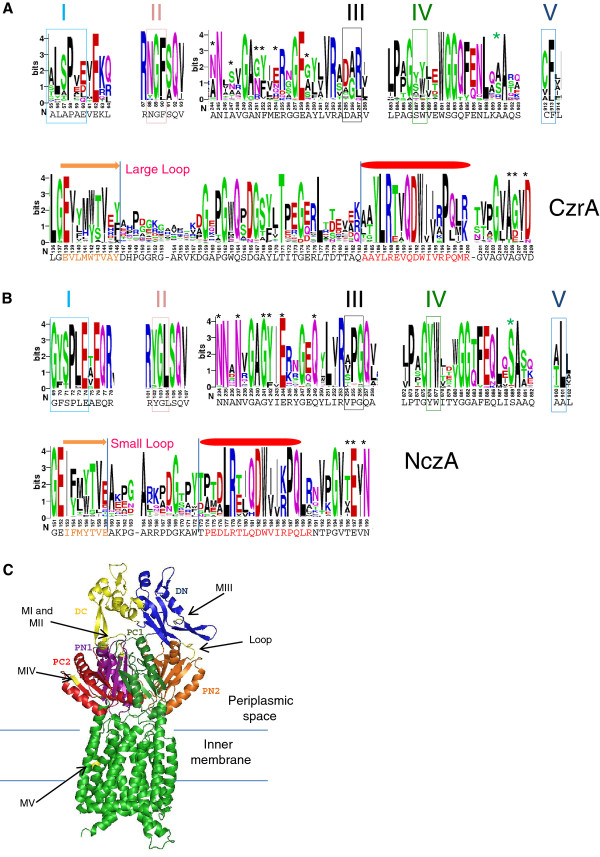
**Motif signatures of the CzrA and NczA orthologous groups and localization on the CzrA structural model.** Main differences in the sequence conservation profile between the CzrA (**A**) and NczA (**B**) orthologous groups are shown. The boxes show the residues important for the respective motifs and the asterisks show differences in the degree of the amino acid conservation between the two orthologous groups. Protein sequences with more than 55% identity to either CzrA or NczA from *C. crescentus* NA1000 were used. The figure was generated using the WebLogo server [42], and the height of the residue symbol indicates the degree of conservation within the ortologous groups. The sequence numbering shown below the alignment corresponds to the respective *C. crescentus* NA1000 proteins. The complete representation of the motifs for the CzrA and NczA orthologous groups are shown in Additional file 2: Figure S1. (**C**) Cartoon representation of the CzrA structure model in which the conserved motifs MI-MV and the Loop are colored in yellow. The sub-domains DC, DN, PC1, PC2, PN1 and PN2 are colored in yellow, blue, dark green, red, violet and orange, respectively. The CzrA structure model was obtained using the Phyre^2^ program with CusA structure as a model (PDB: 3 k07, [25]). The structure was generated using PyMOL [43]. The secondary structure elements indicated were predicted using the PHYRE^2^ program [44]; red ovals and amino acid sequences indicate α-helix; orange arrows and amino acid sequences indicate β-strands.

A previous search for domain signatures for the HME subfamilies identified the consensus sequence DFGX_3_DGAX_3_VEN as characteristic of HME1 and HME2 [14]. We used our alignment of *C. crescentus* CzrA and NczA orthologs in order to identify other possible motif signatures for each group (Figure 6). The analysis of the amino acid conservation profile within the CzrA and NczA orthologous groups showed five main different motif signatures (MI-MV) (Figure 6A-B). In CzrA these motifs are: MI - XLXPXX, MII-NGF, MIII -not conserved, MIV- not conserved and MV- CF. In NczA these motifs are: MI - GY/FSPLE, MII - YGL, MIII- PGQ, MIV - YW and MV- XL. A large loop contains the signature motif GXPGXQXDGX_3_TX_2_GX_2_L, whereas the small loop has motif AX_4_G. The complete analysis of the amino acid conservation for CzrA and NczA is shown in Additional file 2: Figure S1.

In order to localize the identified signatures in the CzrA protein structure, we performed a homology modeling analysis utilizing the structure of *E. coli* CusA as model (PDB: 3 k07), since it is the only metal-transporting RND protein structure so far available in the data bases. All of the motifs described above, with the exception of MV, are located in the periplasmic domain of CzrA structural model (Figure 6C). MV is located in TM8 in CzrA (Figure 6C), which in *E. coli* CusA suffers a significant conformational change when it binds Cu^+^ or Ag^+^, and was proposed to be involved in transmembrane signaling and in initiation of proton translocation across the membrane [25]. MI and MII are located in two close loops in the sub-domain PN1, MIII is located in the sub-domain DN and MIV is located in the sub-domain-PC2 (Figure 6C). The PC2 sub-domain in *E. coli* CusA was proposed to move, creating a cleft between PC1 and PC2 when CusA binds to Cu^+^ or Ag^+^[25].

The most conspicuous difference between the CzrA and NczA groups is the length of the loop located in PN2, called here Large Loop for CzrA and Small loop for NczA. The periplasmic PN2 region is involved in the interaction between *E. coli* CusA and one molecule of the CusB dimer [25,45]. When we superimpose the CzrA model on the CusAB_2_ complex structure (PDBID: 3NE5), the results suggest that the Large Loop could affect the interaction between CzrA and the adaptor protein (not shown). The predicted adaptors for the *C. crescentus* HME-RND systems, CzrB and NczB, share no significant amino acid sequence identity with CusB [45]. Nevertheless, most of the interface residues at the sub-domain DC in CusA involved in the interaction with one molecule of the CusB dimer are conserved in the CzrA and NczA orthologs, although the two residues located in PN2, D155 and R147, are not conserved in members of either group. We have to take into consideration the fact that the structures of the two partners have not been experimentally determined, and therefore one cannot infer how the interaction between the adaptors and the RND proteins takes place.

The metal transport by the CusA efflux pump is mediated by a methionine channel built of four methionine pairs, M410-M501, M486-M403, M391-M1009 and M755-M271 and a fifth cluster made up of three more essential methionines, M672, M573 and M623 [25]. In the CzrA-like and NczA-like ortholog families, methionine is only found at one of the positions that correspond to the methionines responsible for Cu^+^/Ag^+^ transport in CusA [25]. In proteins of both families these positions are occupied by other hydrophobic residues (Table 1). Moreover, of the three residues important for the proton-relay network in *E. coli* CusA, D405, E939 and K984 [25], only one is conserved in the CzrA and NczA orthologs (Table 1). This observation raises the question about whether members of these families use methionine pairs/clusters to bind and export metal ions in a manner similar to that described for CusA. One possibility is that the methionine pairs are constituted by other methionines positioned differently in the *C. crescentus* HME-RND structure. CzrA and NczA have 32 and 23 methionine residues, respectively. We therefore attempted to correlate these methionines in the CzrA structure model (see Additional file 3: Figure S2). There is no methionine pair close to the M271-M755 pair from CusA, but a possible M227-M816 pair exists close to the periplasmic region in the CzrA model. The three essential methionine cluster made up of M672, M573 and M623 in CusA could be correlated with the M695 and M644 pair from CzrA. Furthermore, M695 is in the same structural core than another pair, M141-M320, suggesting that the three essential methionines could be replaced with two methionine pairs, M695-M644 and M141-M320. The M1009-M391 and M403-M486 pairs in CusA could be correlated with M1020-M504 and with a cluster of three methionines (M420, M410 and M403) respectively, in the CzrA model. All of these methionines are located in the transmembrane domain of CusA/CzrA. Nevertheless, there does not seem to be a methionine pair in CzrA that corresponds with M410-M501 in CusA. Methionine pairs in the CzrA transmembrane region with Sδ-Sδ distances greater than 11 Å are M977-M1007, M1000-M1007 and M472-M1008. All of these potential methionine pairs showing some spatial correlation with the CusA methionine pairs/clusters do not form an obvious channel in the CzrA model (Additional file 3: Figure S2D). This could be due to errors in the model which is based on the CusA structure with which it shares only 33% identity and 54% similarity. Another possibility is that members of the CzrA family bind and export divalent ions in a different manner than members of CusA family transport Cu^+^ and Ag^+^ monovalent ions. This could be a reflection of a divergent evolutionary process in which members of CusA and CzrA evolved separately achieving different mechanisms and specificities for metal ions. However the degree of similarities and differences in the transport mechanisms of these two families remains to be established.

**Table 1:** **Correlation between some relevant residues in *E. coli *CusA with the corresponding residues in the CzrA and NczA orthologs**

**CusA**	M271	M391	M403	M410	M486	M501	M573	M623	M672	M755	M1009	D405	E939	K984
**CzrA**	L301^a^	M420	I430	I437	L512	V527	Q598	A648	E699	A782	Q1031	D432	L972	V1006
**NczA**	L291	M410	I420	I427	L502	V517	H588	V638	E689	V722	Q1020	D422	L961	V995
**Residue conserved**^**b**^	--- (L)	***M*** (M)	I/L (I/L)	I/V (I/V)	***L*** (***L***)	---(V/I)	--- (−−-)	--- (−−-)	***E*** (***E***)	--- (−−-)	***Q *( *Q *)**	***D *( *D *)**	***L *( *L *)**	***V***(***V/I***)

^a^The numbers correspond to the positions in *C. crescentus* CzrA and NczA proteins, respectively. The correspondence was determined by sequence alignment made using the PHYRE^2^ program [44].

^b^ Conservation profile within the CzrA-like proteins and in the NczA-like proteins (in parentheses). A conserved residue was considered the residue most prevalent (more than 75% conserved) in that position within the CzcA orthologous groups. In bold and italic are shown the residues absolutely conserved and ----- is not conserved.

## Conclusion

In this work, we show a comparison of two HME-RND family efflux systems (*czrCBA* and *nczCBA*), where the RND proteins (CzrA and NczA) have the motif DFG-GAD-VEN involved in the export of metal divalent cations. Gene expression analyses, as well as metal resistance profile of mutant strains, showed that *czrA* is involved mainly in response to cadmium and zinc with a secondary role in response to cobalt, whereas the *nczA* is involved mainly in response to nickel and cobalt, with a secondary role in response to cadmium and zinc. Phylogenetic analysis of these two RND proteins showed that they group into separate branches, and that CzrA-like proteins (HME2 group) are mainly found in the Alphaproteobacteria, while NczA-like proteins (HME1 group) are more widespread among Proteobacteria. Signature motifs of each group were identified, but no correlation between phylogenetic distribution and the response to different types of metals was observed.

## Methods

### Bacterial strains, plasmids and growth conditions

Bacterial strains and plasmids used in this study are summarized in Table 2. All *C. crescentu*s strains were grown in PYE medium [46] at 30°C with vigorous shaking. When necessary, kanamycin (5 μg/ml), tetracycline (1 μg/ml), nalidixic acid (20 μg/ml) or sucrose (0.2%) were added. Plasmids were propagated in *E. coli* strain DH5α and mobilized into *C. crescentus* by bacterial conjugation using *E. coli* strain S17-1. *E. coli* strains were grown in Lysogeny Broth (LB) medium, supplemented with tetracycline (12.5 μg/ml), kanamycin (50 μg/ml) or ampicillin (100 μg/ml) when required. The genes studied were: *czrA* (CCNA_02805; GenBank: ACL96270) and *nczA* (CCNA_02471; GenBank: YP_002517844).

**Table 2:** Bacteria strains and plasmids used in this study

**Strains or plasmids**	**Characteristics**	**Source or reference**
***Escherichia coli***		
DH5α	Transformation recipient strain	[47]
S17-1λ	Conjugation donor strain	[48]
***C. crescentus***		
NA1000	Also CB15N, synchronizable derivative of wild-type CB15	[49]
MM46	NA1000 Δ*nczA* (ΔCCNA_02473)	This work
MM47	NA1000 Δ*czrA* (ΔCCNA_02809)	This work
MM48	NA1000*ΔczrA*Δ*nczA*	This work
MM46^+^	MM46 *xylX*::*nczA*	This work
MM47^+^	MM47 *xylX*::*czrA*	This work
**Plasmids**		
pGEM-T Easy	Cloning vector; Amp^r^	Promega
pRK*lacZ*290	pRK2-derived vector with a promoterless *lacZ* gene; Tet^r^	[50]
pNPTS138	Suicide vector used for gene disruption containing *oriT* and *sacB*; Kan^r^	D. Alley
pNPT228XNE	*xylX* locus in pNPT228; Kan^r^	[51]

### Cloning of the promoter regions and β-galactosidase activity assays

Regulatory regions upstream of *C. crescentus* NA1000 ORFs CCNA_02805 (between −379 and +75 relative to the ATG), CCNA_02806 (between −374 and +56) and CCNA_02471 (between −675 and +188) were amplified from purified chromosomal DNA by PCR with Platinum *Pfx* DNA polymerase (Life Technologies) and specific primers (Table 3): RND1/RND2 (P*czc1*), RND3/RND4 (P*czc1a*) and RND5/RND6 (P*czc2*). The amplified fragments were cloned into pGEM-T Easy (Promega) and confirmed by DNA sequencing. Each fragment was ligated upstream of the *lacZ* gene on pRK*lacZ*290 and the recombinant plasmids were transferred to *C. crescentus* strain NA1000.

**Table 3:** Primers used in this study

**Nome**	**Sequence (5**^**′**^**- → 3**^**′ **^**)**^**a**^
RND1	G**GAATTC**GCGATTGGCTAACGG
RND2	C**AAGCTT**GACCAACGCAACCAAG
RND3	G**GAATTC**GCCATCTGCGCCAACGATT
RND4	C**AAGCTT**CTCATGAAGCCTAGAG
RND5	**GGGATCC**GCCGGATCCCTCCGATGTGAAGAGG
RND6	**CCTGCAG**CGGACGCCGGCCTCTGCAGCCGC
RND7	C**AAGCTT**CATCCTCACCCTGAGACAA
RND8	G**GAATTC**AGAGATCCAAGATCCTG
RND9	G**GAATTC**GATCTGCCGGTTCGTCCTG
RND10	CG**ACGCGT**TAGCCTCTTTCAATGTGAAGAC
RND11	C**AAGCTT**CTACCAAGGGCGGTCGCAT
RND12	G**GGATCC**TGGTCGCCTCCCTAATGGT
RND13	G**GGATCC**CATTGAGCCTCCGCCAGCT
RND14	CG**ACGCGT**CTATAGTACCATCGCAATAC
RND15	G**ACTAGT**ATGATCGGCAGGATCTTGGAT
RND16	G**ACTAGT**TTAGGCTCCTTGCTCTTGA
RND17	G**GAATTC**ATGCTTGAACGCATCATCGCC
RND18	G**ACTAGT**CTATCGTACCGCCCTGGCTTG

^**a**^ Boldface letters indicate restriction enzyme recognition sites, used for cloning purposes.

Growth phase-dependent promoter activity was measured by β-galactosidase assays [38], from exponential or stationary phase (24 h) cultures grown in PYE-tetracycline. Expression driven from promoters P*czc1* and P*czc2* was also evaluated in the presence of divalent cations (Sigma) at the following concentrations: 10 μM CdCl_2_; 100 μM ZnCl_2_; 100 μM CoCl_2_; or 100 μM NiCl_2_. Cultures grown in PYE-tetracycline at 30°C were diluted to an initial optical density at 600 nm (OD_600_) of 0.1, and the divalent metal was added when they reached OD_600_ 0.5. Aliquots were taken before and at several time points after metal addition and expression was measured by β-galactosidase assays. Statistical treatment of the data was carried out using Student’s T-Test.

### RT-PCR

Total RNA from exponentially growing *C. crescentus* NA1000 cells was extracted by the Trizol method, as described by the manufacturer (Life Technologies). RNA obtained was treated with 0.6 U of RQ1 DNase (Promega) for 30 min at 37°C, followed by phenol extraction and ethanol precipitation, in order to eliminate contaminating genomic DNA. The RNA integrity was assessed by agarose/formaldehyde gel electrophoresis and quantified in a Nanodrop 2000 device (Thermo Scientific). The reactions were performed using primers RND3 and RND4 (located within the coding region of CCNA_02805 and CCNA_02806, respectively). cDNA was synthesized from 0.25 μg of RNA using Super Script™ First Strand Synthesis System (Life Technologies) in a 20 μl final volume, following the manufacturer’s instructions. PCR amplification was performed using 1.2 μg of cDNA as template, 10 pmol each primer, 5% DMSO in a final volume of 25 μl using Taq DNA polymerase (Fermentas). The PCR conditions were: 94°C for 5 min, followed by 30 cycles of 94°C for 30 s, 45°C for 30 s, and 72°C for 1 min, with a final cycle at 72°C for 5 minutes. A negative control reaction was performed as described above, without the addition of reverse transcriptase. The PCR products were analyzed on 1% agarose gel electrophoresis.

### Construction of the *czrA* and *nczA* mutant and complemented strains

In-frame deletions were constructed by allelic exchange using the pNPTS138 suicide vector and *C. crescentus* NA1000 strain. Two genomic regions upstream and downstream of the gene to be deleted were amplified by PCR using *pfx* Platinum DNA polymerase (Life Technologies) and primers RND7/RND8 (785 bp, *HindIII*/*EcoRI*) and RND9/RND10 (752 bp, *EcoRI*/*MluI*) to *czrA* gene and primers RND11/RND12 (870 bp, *HindIII*/*BamHI*) and RND13/RND14 (654 bp, *BamHI*/*MluI*) to *nczA* gene. A terminal adenine was added with Taq DNA Polymerase (Life Technologies) and subsequently the fragments were cloned into vector pGEM-T Easy (Promega). The fragments were cloned *in tandem* into the pNPTS138 vector, the plasmids were transferred to *C. crescentus* strain NA1000 by conjugation with *E. coli* S17-1, and recombinant colonies were selected in PYE-kanamycin plates. A colony containing the integrated plasmid was inoculated in PYE medium without antibiotics for 48 hours, and loss of the plasmid was selected in PYE media containing 3% sucrose. The deletions were confirmed by PCR. Double mutant Δ*czrA*Δ*nczA* was obtained by introducing the pNPTS138 vector containing the 5^′^ and 3^′^-flanking regions of *czrA* into the Δ*nczA* strain. PCR products using primers RND15/RND16 (3243 bp) and RND17/RND18 (3132 bp), containing the coding regions of *czc1* and *czc2* genes respectively, were used to generate complemented strains. Each fragment was cloned into the suicide vector pNPT228XNE, and the plasmid was inserted into the mutant strains by conjugation with *E. coli* S17-1. The insertion of the recombinant vector occurs at the xylose utilization locus, and expression of the cloned genes is induced with 0.2% xylose.

### Growth assays in the presence of metals

Initial cultures at OD_600_ = 0.05 in PYE medium were divided into tubes containing or not each metal (40 μM CdCl_2_, 100 μM ZnCl_2_, 100 μM CoCl_2_ and 300 μM NiCl_2_), and incubated at 30°C for 24 h with agitation. Growth was determined by measuring the OD_600_ of the cultures.

*C. crescentus* NA1000 and mutant strains carrying either the empty vector pNPT228XNE or the vector harbouring either *czrA* or *nczA* genes were grown in PYE-kanamycin at 30°C with agitation to an OD_600_ of 0.3. Samples of 10 μl were streaked on PYE-kanamycin plates containing 2% xylose and with or without addition of each of the following metal salts: 35 μM CdCl_2_, 130 μM ZnCl_2_, 50 μM CoCl_2_ and 280 μM NiCl_2_, and plates were incubated at 30°C for 3 days. Statistical treatment of the data was carried out using Student’s T-Test.

### Phylogenetic and protein structure analyses

Amino acid sequences presenting more than 55% identity with CzrA and NczA were used as an imput for CLUSTALX [40]. The complete list of the protein sequences used is found in Additional file 1: Table S1. The phylogenetic tree was constructed by a neighbor-joining method with 1000 bootstrap replicates using the CLUSTALX program. The multiple sequence alignment was used to create the logo representation of the CzrA and NczA orthologous grups. The figure was generated using the WebLogo server [42] and the height of the residue symbol indicates the degree of conservation. The sequence numbering shown below the logo corresponds to the proteins from *C. crescentus* NA1000.

Homology modeling of CzrA was performed using the PHYRE2 [44] using as a three-dimensional structural template the chain A of *E. coli* CusA [PDB: 3 k07; [25]. CzrA and CusA share 33% sequence identity. The model generated has 100% confidence and 93% coverage. The result was analyzed with the PyMOL Molecular Graphics System, Version 1.5 Schrödinger, LLC [43].

## Competing interest

The authors declare that they have no competing interests.

## Authors’ contributions

EYV designed and performed the experimental work and drafted the manuscript. VSB participated in the design of the study and performed some of the expression assays. CG did the protein structure modeling and analysis. MVM conceived the study, and participated in its design, coordination and helped to draft the manuscript. All authors read and approved the final manuscript.

## Supplementary Material

Additional file 1: Table S1 Protein sequences used for the phylogenetic analysis of the HME-RND orthologs. Click here for file

Additional file 2: Figure S1 Sequence conservation profile within the CzrA and NczA orthologous groups. Click here for file

Additional file 3: Figure S2 Potential methionine pairs/clusters in CzrA model structure. Click here for file
