# Supplementary material for: Two RND proteins involved in heavy metal efflux in Caulobacter crescentus belong to separate clusters within proteobacteria
Source: BMC Microbiol. 2013 Apr 11;13:79. doi: 10.1186/1471-2180-13-79 (PMC3637150; doi:10.1186/1471-2180-13-79)
Supplement: Additional file 1: Table S1 — Protein sequences used for the phylogenetic analysis of the HME-RND orthologs. [file 1471-2180-13-79-S1.pdf]

Valencia E. Y., Braz, V. S., Guzzo, C. and Marques, M. V. Two RND proteins involved in heavy metal efflux in *Caulobacter crescentus* belong to separate clusters within Proteobacteria

Supplemental Table 1: Protein sequences used for the phylogenetic analysis of the HME-RND orthologs

>gi|103487928|ref|YP_617489.1| heavy metal efflux pump CzcA [Sphingopyxis alaskensis RB2256]

>gi|103488047|ref|YP_617608.1| heavy metal efflux pump CzcA [Sphingopyxis alaskensis RB2256]

>gi|103488057|ref|YP_617618.1| heavy metal efflux pump CzcA [Sphingopyxis alaskensis RB2256]

>gi|104781163|ref|YP_607661.1| RND divalent metal cation efflux transporter CzcA [Pseudomonas entomophila L48]

>gi|107027026|ref|YP_624537.1| heavy metal efflux pump CzcA [Burkholderia cenocepacia AU 1054]

>gi|109898348|ref|YP_661603.1| CzcA family heavy metal efflux protein [Pseudoalteromonas atlantica T6c]

>gi|110834238|ref|YP_693097.1| czcA1 gene product [Alcanivorax borkumensis SK2]

>gi|110834244|ref|YP_693103.1| czcA3 gene product [Alcanivorax borkumensis SK2]

>gi|114330498|ref|YP_746720.1| heavy metal efflux pump, CzcA family protein [Nitrosomonas eutropha C91]

>gi|114564613|ref|YP_752127.1| heavy metal efflux pump, CzcA family protein [Shewanella frigidimarina NCIMB 400]

>gi|116050541|ref|YP_790640.1| cation efflux system protein [Pseudomonas aeruginosa UCBPP-PA14]

>gi|116696343|ref|YP_841919.1| cation/multidrug efflux pump [Ralstonia eutropha H16]

>gi|117676094|ref|YP_863670.1| CzcA family heavy metal efflux protein [Shewanella sp. ANA-3]

>gi|117676321|ref|YP_863897.1| CzcA family heavy metal efflux protein [Shewanella sp. ANA-3]

>gi|119468867|ref|ZP_01611892.1| Cobalt-zinc-cadmium resistance protein czcA [Alteromonadales bacterium TW-7]

>gi|120597183|ref|YP_961757.1| CzcA family heavy metal efflux protein [Shewanella sp. W3-18-1]

>gi|120597988|ref|YP_962562.1| CzcA family heavy metal efflux protein [Shewanella sp. W3-18-1]

>gi|120611175|ref|YP_970853.1| CzcA family heavy metal efflux protein [Acidovorax citrulli AAC00-1]

>gi|121594215|ref|YP_986111.1| CzcA family heavy metal efflux protein [Acidovorax sp. JS42]

>gi|121597208|ref|YP_989628.1| heavy metal efflux pump CzcA [Burkholderia mallei SAVP1]

>gi|124267544|ref|YP_001021548.1| RND OM permease [Methylibium petroleiphilum PM1]

>gi|12484566|gb|AAG09629.1| CztA [Pseudomonas fluorescens]

>gi|126442386|ref|YP_001062521.1| CzcA family heavy metal efflux protein [Burkholderia pseudomallei 668]

>gi|126643226|ref|YP_001086210.1| RND divalent metal cation efflux transporter [Acinetobacter baumannii ATCC 17978]

>gi|134093787|ref|YP_001098862.1| czcA gene product [Herminiimonas arsenicoxydans]

>gi|134094813|ref|YP_001099888.1| czcA gene product [Herminiimonas arsenicoxydans]

>gi|134094822|ref|YP_001099897.1| czcA gene product [Herminiimonas arsenicoxydans]

>gi|134283500|ref|ZP_01770200.1| heavy metal efflux pump CzcA [Burkholderia pseudomallei 305]

>gi|14132747|gb|AAK52285.1| membrane-bound cation-proton-antiporter CzrA [Pseudomonas putida]

>gi|141924|gb|AAA21970.1| transmembrane transporter [Cupriavidus metallidurans CH34]

>gi|146283742|ref|YP_001173895.1| cation efflux family protein [Pseudomonas stutzeri A1501]

>gi|146291336|ref|YP_001181760.1| CzcA family heavy metal efflux protein [Shewanella putrefaciens CN-32]

>gi|146293065|ref|YP_001183489.1| CzcA family heavy metal efflux protein [Shewanella putrefaciens CN-32]

>gi|146295073|ref|YP_001185497.1| CzcA family heavy metal efflux protein [Shewanella putrefaciens CN-32]

>gi|146306209|ref|YP_001186674.1| CzcA family heavy metal efflux protein [Pseudomonas mendocina ymp]

>gi|148254829|ref|YP_001239414.1| RND divalent metal cation efflux transporter CnrA [Bradyrhizobium sp. BTAi1]

>gi|148258382|ref|YP_001242967.1| heavy metal efflux pump CzcA [Bradyrhizobium sp. BTAi1]

>gi|148258461|ref|YP_001243046.1| RND divalent metal cation efflux transporter CzcA [Bradyrhizobium sp. BTAi1]

>gi|148359918|ref|YP_001251125.1| cobalt/zinc/cadmium efflux RND transporter permease HelA [Legionella pneumophila str. Corby]

>gi|148360331|ref|YP_001251538.1| hypothetical protein LPC_2269 [Legionella pneumophila str. Corby]

>gi|148548493|ref|YP_001268595.1| CzcA family heavy metal efflux protein [Pseudomonas putida F1]

>gi|148555521|ref|YP_001263103.1| CzcA family heavy metal efflux protein [Sphingomonas wittichii RW1]

>gi|149186552|ref|ZP_01864864.1| metal ion efflux RND protein family protein [Erythrobacter sp. SD-21]

>gi|149926511|ref|ZP_01914772.1| heavy metal efflux pump CzcA [Limnobacter sp. MED105]

>gi|152981249|ref|YP_001353482.1| CzcA family heavy metal efflux protein [Janthinobacterium sp. Marseille]

>gi|152982936|ref|YP_001353422.1| cation efflux system protein [Janthinobacterium sp. Marseille]

>gi|152982939|ref|YP_001353459.1| CzcA family cobalt/zinc/cadmium efflux transporter permease [Janthinobacterium sp. Marseille]

>gi|152989422|ref|YP_001348077.1| RND divalent metal cation efflux transporter CzcA [Pseudomonas aeruginosa PA7]

>gi|154244567|ref|YP_001415525.1| CzcA family heavy metal efflux protein [Xanthobacter autotrophicus Py2]

>gi|154247907|ref|YP_001418865.1| CzcA family heavy metal efflux protein [Xanthobacter autotrophicus Py2]

>gi|154253806|ref|YP_001414630.1| CzcA family heavy metal efflux protein [Parvibaculum lavamentivorans DS-1]

>gi|15597716|ref|NP_251210.1| czcA gene product [Pseudomonas aeruginosa PAO1]

>gi|157377000|ref|YP_001475600.1| cobalt-zinc-cadmium resistance protein CzcA [Shewanella sediminis HAW-EB3]

>gi|157960216|ref|YP_001500250.1| CzcA family heavy metal efflux protein [Shewanella pealeana ATCC 700345]

>gi|159528137|ref|YP_001542700.1| HelA protein [Fluoribacter dumoffii Tex-KL]

>gi|160896535|ref|YP_001562117.1| CzcA family heavy metal efflux protein [Delftia acidovorans SPH-1]

>gi|160897162|ref|YP_001562744.1| CzcA family heavy metal efflux protein [Delftia acidovorans SPH-1]

>gi|16126629|ref|NP_421193.1| AcrB/AcrD/AcrF family protein [Caulobacter crescentus CB15]

>gi|16126957|ref|NP_421521.1| RND protein family metal ion efflux [Caulobacter crescentus CB15]

>gi|161506504|ref|YP_001573625.1| CzcA family heavy metal efflux protein [Burkholderia multivorans ATCC 17616]

>gi|162456995|ref|YP_001619362.1| czcA2 gene product [Sorangium cellulosum 'So ce 56']

>gi|163858926|ref|YP_001633224.1| czcA gene product [Bordetella petrii DSM 12804]

>gi|167031078|ref|YP_001666309.1| CzcA family heavy metal efflux protein [Pseudomonas putida GB-1]

>gi|167033050|ref|YP_001668281.1| CzcA family heavy metal efflux protein [Pseudomonas putida GB-1]

>gi|167565734|ref|ZP_02358650.1| heavy metal efflux pump CzcA [Burkholderia oklahomensis EO147]

>gi|167572830|ref|ZP_02365704.1| heavy metal efflux pump CzcA [Burkholderia oklahomensis C6786]

>gi|167577989|ref|ZP_02370863.1| heavy metal efflux pump CzcA [Burkholderia thailandensis TXDOH]

>gi|167590139|ref|ZP_02382527.1| heavy metal efflux pump, CzcA family protein [Burkholderia ubonensis Bu]

>gi|167625809|ref|YP_001676103.1| CzcA family heavy metal efflux protein [Shewanella halifaxensis HAW-EB4]

>gi|167646248|ref|YP_001683911.1| CzcA family heavy metal efflux protein [Caulobacter sp. K31]

>gi|167646259|ref|YP_001683922.1| CzcA family heavy metal efflux protein [Caulobacter sp. K31]

>gi|167723509|ref|ZP_02406745.1| heavy metal efflux pump CzcA [Burkholderia pseudomallei DM98]

>gi|167742475|ref|ZP_02415249.1| heavy metal efflux pump CzcA [Burkholderia pseudomallei 14]

>gi|167819644|ref|ZP_02451324.1| heavy metal efflux pump CzcA [Burkholderia pseudomallei 91]

>gi|167828027|ref|ZP_02459498.1| heavy metal efflux pump CzcA [Burkholderia pseudomallei 9]

>gi|167839647|ref|ZP_02466331.1| heavy metal efflux pump CzcA [Burkholderia thailandensis MSMB43]

>gi|167906420|ref|ZP_02493625.1| heavy metal efflux pump CzcA [Burkholderia pseudomallei NCTC 13177]

>gi|167914755|ref|ZP_02501846.1| heavy metal efflux pump CzcA [Burkholderia pseudomallei 112]

>gi|169632229|ref|YP_001705965.1| RND divalent metal cation efflux transporter [Acinetobacter baumannii SDF]

>gi|169794463|ref|YP_001712256.1| RND divalent metal cation efflux transporter [Acinetobacter baumannii AYE]

>gi|169883788|gb|ACA97990.1| CzrA [Pseudomonas aeruginosa]

>gi|170725032|ref|YP_001759058.1| CzcA family heavy metal efflux protein [Shewanella woodyi ATCC 51908]

>gi|170736224|ref|YP_001777484.1| CzcA family heavy metal efflux protein [Burkholderia cenocepacia MC0-3]

>gi|170747820|ref|YP_001754080.1| CzcA family heavy metal efflux protein [Methylobacterium radiotolerans JCM 2831]

>gi|1731918|emb|CAA67084.1| membrane-bound cation-proton-antiporter [Cupriavidus metallidurans CH34]

>gi|17548714|ref|NP_522054.1| czcA gene product [Ralstonia solanacearum GMI1000]

>gi|184159730|ref|YP_001848069.1| putative silver efflux pump [Acinetobacter baumannii ACICU]

>gi|187928845|ref|YP_001899332.1| CzcA family heavy metal efflux pump [Ralstonia pickettii 12J]

>gi|188583291|ref|YP_001926736.1| CzcA family heavy metal efflux pump [Methylobacterium populi BJ001]

>gi|188591817|ref|YP_001796416.1| Cobalt-zinc-cadmium resistance membrane component, Cation efflux system [Cupriavidus taiwanensis]

>gi|190572123|ref|YP_001969968.1| putative AcrA/AcrD/AcrF family protein [Stenotrophomonas maltophilia K279a]

>gi|190574393|ref|YP_001972238.1| putative Cobalt-zinc-cadmium resistance protein [Stenotrophomonas maltophilia K279a]

>gi|192290688|ref|YP_001991293.1| CzcA family heavy metal efflux pump [Rhodopseudomonas palustris TIE-1]

>gi|192290731|ref|YP_001991336.1| CzcA family heavy metal efflux pump [Rhodopseudomonas palustris TIE-1]

>gi|192359173|ref|YP_001984146.1| CzcA family heavy metal efflux pump [Cellvibrio japonicus Ueda107]

>gi|192361483|ref|YP_001982318.1| CzcA family heavy metal efflux pump [Cellvibrio japonicus Ueda107]

>gi|193078580|gb|ABO13608.2| RND divalent metal cation efflux transporter [Acinetobacter baumannii ATCC 17978]

>gi|194292893|ref|YP_002008800.1| czcA1 gene product [Cupriavidus taiwanensis LMG 19424]

>gi|197103380|ref|YP_002128758.1| heavy metal efflux pump CzcA [Phenylobacterium zucineum HLK1]

>gi|197103396|ref|YP_002128774.1| heavy metal efflux pump CzcA [Phenylobacterium zucineum HLK1]

>gi|197104898|ref|YP_002130275.1| cation efflux system membrane protein A [Phenylobacterium zucineum HLK1]

>gi|197104911|ref|YP_002130288.1| AcrB/AcrD/AcrF family protein [Phenylobacterium zucineum HLK1]

>gi|206562573|ref|YP_002233336.1| putative cobalt-zinc-cadmium resistance efflux system transporter protein [Burkholderia cenocepacia J2315]

>gi|206589467|emb|CAQ36428.1| cation efflux system protein [Ralstonia solanacearum MolK2]

>gi|209883889|ref|YP_002287746.1| cation efflux system protein CzcA [Oligotropha carboxidovorans OM5]

>gi|209885558|ref|YP_002289415.1| cation efflux system protein CzcA [Oligotropha carboxidovorans OM5]

>gi|209886831|ref|YP_002290688.1| cation efflux system protein CzcA [Oligotropha carboxidovorans OM5]

>gi|209886842|ref|YP_002290699.1| CzcA family heavy metal efflux pump [Oligotropha carboxidovorans OM5]

>gi|21233458|ref|NP_639375.1| cation efflux system protein [Xanthomonas campestris pv. campestris str. ATCC 33913]

>gi|21244877|ref|NP_644459.1| czcA gene product [Xanthomonas axonopodis pv. citri str. 306]

>gi|212635881|ref|YP_002312406.1| cobalt-zinc-cadmium resistance protein CzcA [Shewanella piezotolerans WP3]

>gi|212635959|ref|YP_002312484.1| heavy metal efflux pump CzcA [Shewanella piezotolerans WP3]

>gi|213967851|ref|ZP_03395998.1| cation efflux family protein [Pseudomonas syringae pv. tomato T1]

>gi|217418454|ref|ZP_03449961.1| heavy metal efflux pump CzcA [Burkholderia pseudomallei 576]

>gi|218529086|ref|YP_002419902.1| CzcA family heavy metal efflux pump [Methylobacterium chloromethanicum CM4]

>gi|220922123|ref|YP_002497424.1| CzcA family heavy metal efflux pump [Methylobacterium nodulans ORS 2060]

>gi|221064871|ref|ZP_03540976.1| heavy metal efflux pump, CzcA family [Comamonas testosteroni KF-1]

>gi|221067035|ref|ZP_03543140.1| heavy metal efflux pump, CzcA family [Comamonas testosteroni KF-1]

>gi|221133393|ref|ZP_03559698.1| CzcA family heavy metal efflux protein [Glaciecola sp. HTCC2999]

>gi|221133583|ref|ZP_03559888.1| CzcA family heavy metal efflux protein [Glaciecola sp. HTCC2999]

>gi|222111387|ref|YP_002553651.1| CzcA family heavy metal efflux pump [Acidovorax ebreus TPSY]

>gi|226943001|ref|YP_002798074.1| heavy metal efflux pump CzcA family [Azotobacter vinelandii DJ]

>gi|226951244|ref|ZP_03821708.1| cobalt-zinc-cadmium resistance membrane component, cation efflux system [Acinetobacter sp. ATCC 27244]

>gi|226953720|ref|ZP_03824184.1| RND divalent metal cation efflux transporter [Acinetobacter sp. ATCC 27244]

>gi|229588247|ref|YP_002870366.1| cobalt-zinc-cadmium resistance membrane protein [Pseudomonas fluorescens SBW25]

>gi|237508857|ref|ZP_04521572.1| cation efflux system protein CzcA [Burkholderia pseudomallei MSHR346]

>gi|239501986|ref|ZP_04661296.1| RND divalent metal cation efflux transporter [Acinetobacter baumannii AB900]

>gi|239503298|ref|ZP_04662608.1| heavy metal efflux pump, CzcA family protein [Acinetobacter baumannii AB900]

>gi|239816528|ref|YP_002945438.1| CzcA family heavy metal efflux pump [Variovorax paradoxus S110]

>gi|239996664|ref|ZP_04717188.1| CzcA family heavy metal efflux protein [Alteromonas macleodii ATCC 27126]

>gi|240111957|ref|YP_002961234.1| RND divalent metal cation efflux transporter CzcA [Methylobacterium extorquens AM1]

>gi|240139227|ref|YP_002963702.1| czcA gene product [Methylobacterium extorquens AM1]

>gi|241114121|ref|YP_002973596.1| heavy metal efflux pump, CzcA family [Ralstonia pickettii 12D]

>gi|241114136|ref|YP_002973611.1| heavy metal efflux pump, CzcA family [Ralstonia pickettii 12D]

>gi|2498437|sp|Q48815.1|HELA_LEGPN RecName: Full=Protein HelA

>gi|253996846|ref|YP_003048910.1| CzcA family heavy metal efflux pump [Methylotenera mobilis JLW8]

>gi|254240951|ref|ZP_04934273.1| Resistance-Nodulation-Cell Division (RND) divalent metal cation efflux transporter CzcA [Pseudomonas aeruginosa 2192]

>gi|254248974|ref|ZP_04942294.1| hypothetical protein BCPG_03829 [Burkholderia cenocepacia PC184]

>gi|254420352|ref|ZP_05034076.1| heavy metal efflux pump, CzcA family [Brevundimonas sp. BAL3]

>gi|254428381|ref|ZP_05042088.1| heavy metal efflux pump, CzcA family [Alcanivorax sp. DG881]

>gi|254448033|ref|ZP_05061497.1| cation efflux system protein CzcA [gamma proteobacterium HTCC5015]

>gi|254448235|ref|ZP_05061697.1| heavy metal efflux pump CzcA [gamma proteobacterium HTCC5015]

>gi|254490348|ref|ZP_05103537.1| heavy metal efflux pump, CzcA family [Methylophaga thiooxidans DMS010]

>gi|254490580|ref|ZP_05103766.1| heavy metal efflux pump, CzcA family [Methylophaga thiooxidans DMS010]

>gi|254521300|ref|ZP_05133355.1| heavy metal efflux pump, CzcA family [Stenotrophomonas sp. SKA14]

>gi|254522678|ref|ZP_05134733.1| heavy metal efflux pump, CzcA family [Stenotrophomonas sp. SKA14]

>gi|254558959|ref|YP_003066054.1| cation efflux system protein czcA [Methylobacterium extorquens DM4]

>gi|254559738|ref|YP_003066833.1| cobalt-zinc-cadmium resistance protein czcA [Methylobacterium extorquens DM4]

>gi|254559901|ref|YP_003066996.1| cation efflux system protein czcA [Methylobacterium extorquens DM4]

>gi|254786127|ref|YP_003073556.1| heavy metal efflux pump, CzcA family [Teredinibacter turnerae T7901]

>gi|255319113|ref|ZP_05360334.1| cation efflux system protein CzcA [Acinetobacter radioresistens SK82]

>gi|255320089|ref|ZP_05361282.1| heavy metal efflux pump, CzcA family [Acinetobacter radioresistens SK82]

>gi|256822500|ref|YP_003146463.1| CzcA family heavy metal efflux pump [Kangiella koreensis DSM 16069]

>gi|257455085|ref|ZP_05620325.1| cation efflux system protein CzcA [Enhydrobacter aerosaccus SK60]

>gi|257482934|ref|ZP_05636975.1| cation efflux family protein [Pseudomonas syringae pv. tabaci str. ATCC 11528]

>gi|260550813|ref|ZP_05825020.1| RND divalent metal cation efflux transporter [Acinetobacter sp. RUH2624]

>gi|260556896|ref|ZP_05829113.1| RND divalent metal cation efflux transporter [Acinetobacter baumannii ATCC 19606]

>gi|262089631|gb|ACY24728.1| CzcA cation efflux system protein [uncultured organism]

>gi|262280361|ref|ZP_06058145.1| RND divalent metal cation efflux transporter [Acinetobacter calcoaceticus RUH2202]

>gi|262368275|ref|ZP_06061604.1| cobalt-zinc-cadmium resistance membrane component [Acinetobacter johnsonii SH046]

>gi|262368292|ref|ZP_06061621.1| cation efflux system protein czcA [Acinetobacter johnsonii SH046]

>gi|262371238|ref|ZP_06064558.1| cation efflux system protein [Acinetobacter johnsonii SH046]

>gi|262373657|ref|ZP_06066935.1| cation efflux system protein czcA [Acinetobacter junii SH205]

>gi|262377596|ref|ZP_06070817.1| cation efflux system protein czcA [Acinetobacter lwoffii SH145]

>gi|262378146|ref|ZP_06071303.1| RND divalent metal cation efflux transporter [Acinetobacter radioresistens SH164]

>gi|264680514|ref|YP_003280424.1| cobalt-zinc-cadmium resistance protein CzcA [Comamonas testosteroni CNB-2]

>gi|26986788|ref|NP_742213.1| CzcA family cobalt/zinc/cadmium efflux transporter permease [Pseudomonas putida KT2440]

>gi|26989133|ref|NP_744558.1| cobalt-zinc-cadmium resistance protein CzcA [Pseudomonas putida KT2440]

>gi|270158441|ref|ZP_06187098.1| HelA protein [Legionella longbeachae D-4968]

>gi|27380048|ref|NP_771577.1| cation efflux system protein [Bradyrhizobium japonicum USDA 110]

>gi|282890304|ref|ZP_06298833.1| hypothetical protein pah_c015o007 [Parachlamydia acanthamoebae str. Hall's coccus]

>gi|282892391|ref|ZP_06300741.1| hypothetical protein pah_c253o005 [Parachlamydia acanthamoebae str. Hall's coccus]

>gi|28867605|ref|NP_790224.1| cation efflux family protein [Pseudomonas syringae pv. tomato str. DC3000]

>gi|289623880|ref|ZP_06456834.1| cation efflux family protein [Pseudomonas syringae pv. aesculi str. NCPPB 3681]

>gi|289646925|ref|ZP_06478268.1| cation efflux family protein [Pseudomonas syringae pv. aesculi str. 2250]

>gi|289666102|ref|ZP_06487683.1| cation efflux system protein [Xanthomonas campestris pv. vasculorum NCPPB 702]

>gi|289670771|ref|ZP_06491846.1| cation efflux system protein [Xanthomonas campestris pv. musacearum NCPPB 4381]

>gi|292491728|ref|YP_003527167.1| CzcA family heavy metal efflux pump [Nitrosococcus halophilus Nc4]

>gi|292491760|ref|YP_003527199.1| CzcA family heavy metal efflux pump [Nitrosococcus halophilus Nc4]

>gi|293602284|ref|ZP_06684731.1| cation efflux system protein CzcA [Achromobacter piechaudii ATCC 43553]

>gi|293610690|ref|ZP_06692990.1| conserved hypothetical protein [Acinetobacter sp. SH024]

>gi|294140993|ref|YP_003556971.1| CzcA family heavy metal efflux pump [Shewanella violacea DSS12]

>gi|294626429|ref|ZP_06705029.1| cation efflux system protein [Xanthomonas fuscans subsp. aurantifolii str. ICPB 11122]

>gi|294651795|ref|ZP_06729092.1| cobalt-zinc-cadmium resistance protein CzcA [Acinetobacter haemolyticus ATCC 19194]

>gi|294666228|ref|ZP_06731481.1| cation efflux system protein [Xanthomonas fuscans subsp. aurantifolii str. ICPB 10535]

>gi|295689771|ref|YP_003593464.1| CzcA family heavy metal efflux pump [Caulobacter segnis ATCC 21756]

>gi|295689893|ref|YP_003593586.1| CzcA family heavy metal efflux pump [Caulobacter segnis ATCC 21756]

>gi|296106647|ref|YP_003618347.1| helA cobalt/zinc/cadmium efflux RND transporter, permease protein HelA [Legionella pneumophila 2300/99 Alcoy]

>gi|296284632|ref|ZP_06862630.1| metal ion efflux RND protein family protein [Citromicrobium bathyomarinum JL354]

>gi|296445159|ref|ZP_06887119.1| heavy metal efflux pump, CzcA family [Methylosinus trichosporium OB3b]

>gi|296532641|ref|ZP_06895340.1| metal ion efflux RND protein [Roseomonas cervicalis ATCC 49957]

>gi|298489235|ref|ZP_07007253.1| Cobalt-zinc-cadmium resistance protein czcA; Cation efflux system protein cusA [Pseudomonas savastanoi pv. savastanoi NCPPB 3335]

>gi|298682178|gb|ADI95246.1| CzcA [Pseudomonas putida DOT-T1E]

>gi|299068880|emb|CBJ40121.1| Cation efflux system protein, heavy metal resistance [Ralstonia solanacearum CMR15]

>gi|299073790|emb|CBJ53311.1| Cation efflux system protein, heavy metal resistance [Ralstonia solanacearum CFBP2957]

>gi|299132562|ref|ZP_07025757.1| heavy metal efflux pump, CzcA family [Afipia sp. 1NLS2]

>gi|299133946|ref|ZP_07027140.1| heavy metal efflux pump, CzcA family [Afipia sp. 1NLS2]

>gi|299532925|ref|ZP_07046312.1| Co/Zn/Cd efflux pump protein CzcA [Comamonas testosteroni S44]

>gi|299768525|ref|YP_003730551.1| putative silver efflux pump [Acinetobacter oleivorans DR1]

>gi|300021966|ref|YP_003754577.1| CzcA family heavy metal efflux pump [Hyphomicrobium denitrificans ATCC 51888]

>gi|300114297|ref|YP_003760872.1| CzcA family heavy metal efflux pump [Nitrosococcus watsonii C-113]

>gi|300313716|ref|YP_003777808.1| cobalt-zinc-cadmium resistance cation efflux system transmembrane protein [Herbaspirillum seropedicae SmR1]

>gi|300693445|ref|YP_003749418.1| cation efflux system protein, heavy metal resistance [Ralstonia solanacearum PSI07]

>gi|302186895|ref|ZP_07263568.1| heavy metal efflux pump CzcA [Pseudomonas syringae pv. syringae 642]

>gi|30249602|ref|NP_841672.1| acriflavin resistance protein:heavy metal efflux pump CzcA [Nitrosomonas europaea ATCC 19718]

>gi|302878162|ref|YP_003846726.1| CzcA family heavy metal efflux pump [Gallionella capsiferriformans ES-2]

>gi|304310142|ref|YP_003809740.1| acrB gene product [gamma proteobacterium HdN1]

>gi|304322325|ref|YP_003855968.1| AcrB/AcrD/AcrF family protein [Parvularcula bermudensis HTCC2503]

>gi|307610899|emb|CBX00516.1| HelA protein [Legionella pneumophila 130b]

>gi|308049362|ref|YP_003912928.1| CzcA family heavy metal efflux pump [Ferrimonas balearica DSM 9799]

>gi|309778698|ref|ZP_07673472.1| cobalt-zinc-cadmium resistance protein CzcA [Ralstonia sp. 5_7_47FAA]

>gi|311107852|ref|YP_003980705.1| cation efflux system protein CZCA [Achromobacter xylosoxidans A8]

>gi|313107416|ref|ZP_07793607.1| Resistance-Nodulation-Cell Division (RND) divalent metal cation efflux transporter CzcA [Pseudomonas aeruginosa 39016]

>gi|315127540|ref|YP_004069543.1| cobalt-zinc-cadmium resistance protein CzcA [Pseudoalteromonas sp. SM9913]

>gi|315127687|ref|YP_004069690.1| heavy metal efflux pump CzcA [Pseudoalteromonas sp. SM9913]

>gi|315500813|ref|YP_004089614.1| heavy metal efflux pump, CzcA family [Asticcacaulis excentricus CB 48]

>gi|316934771|ref|YP_004109753.1| CzcA family heavy metal efflux pump [Rhodopseudomonas palustris DX-1]

>gi|317404061|gb|EFV84518.1| cobalt-zinc-cadmium resistance protein czcA [Achromobacter xylosoxidans C54]

>gi|319760956|ref|YP_004124893.1| heavy metal efflux pump, czca family [Alicycliphilus denitrificans BC]

>gi|319794721|ref|YP_004156361.1| CzcA family heavy metal efflux pump [Variovorax paradoxus EPS]

>gi|320326319|gb|EFW82372.1| cation efflux family protein [Pseudomonas syringae pv. glycinea str. B076]

>gi|320331688|gb|EFW87626.1| cation efflux family protein [Pseudomonas syringae pv. glycinea str. race 4]

>gi|323135879|ref|ZP_08070962.1| heavy metal efflux pump, CzcA family [Methylocystis sp. ATCC 49242]

>gi|325272079|ref|ZP_08138515.1| CzcA family heavy metal efflux protein [Pseudomonas sp. TJI-51]

>gi|325273031|ref|ZP_08139342.1| CzcA family heavy metal efflux protein [Pseudomonas sp. TJI-51]

>gi|325916748|ref|ZP_08179002.1| heavy metal efflux pump, cobalt-zinc-cadmium [Xanthomonas vesicatoria ATCC 35937]

>gi|325918043|ref|ZP_08180204.1| heavy metal efflux pump, cobalt-zinc-cadmium [Xanthomonas vesicatoria ATCC 35937]

>gi|325922640|ref|ZP_08184387.1| heavy metal efflux pump, cobalt-zinc-cadmium [Xanthomonas gardneri ATCC 19865]

>gi|325923805|ref|ZP_08185420.1| heavy metal efflux pump, cobalt-zinc-cadmium [Xanthomonas gardneri ATCC 19865]

>gi|325925990|ref|ZP_08187356.1| heavy metal efflux pump, cobalt-zinc-cadmium [Xanthomonas perforans 91-118]

>gi|325981358|ref|YP_004293760.1| CzcA family heavy metal efflux pump [Nitrosomonas sp. AL212]

>gi|325983726|ref|YP_004296127.1| CzcA family heavy metal efflux pump [Nitrosomonas sp. AL212]

>gi|326317516|ref|YP_004235188.1| CzcA family heavy metal efflux pump [Acidovorax avenae subsp. avenae ATCC 19860]

>gi|326388731|ref|ZP_08210320.1| metal ion efflux RND protein family protein [Novosphingobium nitrogenifigens DSM 19370]

>gi|329848011|ref|ZP_08263039.1| cation efflux system protein czcA [Asticcacaulis biprosthecum C19]

>gi|329889061|ref|ZP_08267404.1| nickel and cobalt resistance protein cnrA [Brevundimonas diminuta ATCC 11568]

>gi|329898634|ref|ZP_08272425.1| Cobalt-zinc-cadmium resistance protein CzcA; Cation efflux system protein CusA [gamma proteobacterium IMCC3088]

>gi|329910801|ref|ZP_08275386.1| Cobalt-zinc-cadmium resistance protein CzcA; Cation efflux system protein CusA [Oxalobacteraceae bacterium IMCC9480]

>gi|330825946|ref|YP_004389249.1| CzcA family heavy metal efflux pump [Alicycliphilus denitrificans K601]

>gi|330891390|gb|EGH24051.1| cation efflux family protein [Pseudomonas syringae pv. mori str. 301020]

>gi|330955033|gb|EGH55293.1| heavy metal efflux pump CzcA [Pseudomonas syringae Cit 7]

>gi|330961962|gb|EGH62222.1| cation efflux family protein [Pseudomonas syringae pv. maculicola str. ES4326]

>gi|330967261|gb|EGH67521.1| cation efflux family protein [Pseudomonas syringae pv. actinidiae str. M302091]

>gi|330972953|gb|EGH73019.1| heavy metal efflux pump CzcA [Pseudomonas syringae pv. aceris str. M302273]

>gi|330989500|gb|EGH87603.1| cation efflux family protein [Pseudomonas syringae pv. lachrymans str. M301315]

>gi|331011821|gb|EGH91877.1| cation efflux family protein [Pseudomonas syringae pv. tabaci str. ATCC 11528]

>gi|331014911|gb|EGH94967.1| cation efflux family protein [Pseudomonas syringae pv. lachrymans str. M302278]

>gi|332139707|ref|YP_004425445.1| Heavy metal efflux pump CzcA [Alteromonas macleodii str. 'Deep ecotype']

>gi|332139772|ref|YP_004425510.1| putative RND efflux system protein [Alteromonas macleodii str. 'Deep ecotype']

>gi|332141453|ref|YP_004427191.1| putative RND efflux system protein [Alteromonas macleodii str. 'Deep ecotype']

>gi|332188600|ref|ZP_08390318.1| heavy metal efflux pump, CzcA family protein [Sphingomonas sp. S17]

>gi|332188604|ref|ZP_08390322.1| heavy metal efflux pump, CzcA family protein [Sphingomonas sp. S17]

>gi|332308751|ref|YP_004436601.1| CzcA family heavy metal efflux pump [Glaciecola sp. 4H-3-7+YE-5]

>gi|332308765|ref|YP_004436615.1| CzcA family heavy metal efflux pump [Glaciecola sp. 4H-3-7+YE-5]

>gi|332308783|ref|YP_004436633.1| CzcA family heavy metal efflux pump [Glaciecola sp. 4H-3-7+YE-5]

>gi|332534830|ref|ZP_08410654.1| cobalt-zinc-cadmium resistance protein CzcA [Pseudoalteromonas haloplanktis ANT/505]

>gi|332535291|ref|ZP_08411092.1| cobalt-zinc-cadmium resistance protein CzcA [Pseudoalteromonas haloplanktis ANT/505]

>gi|332873754|ref|ZP_08441696.1| cobalt-zinc-cadmium resistance protein CzcA [Acinetobacter baumannii 6014059]

>gi|333892934|ref|YP_004466809.1| CzcA family heavy metal efflux protein [Alteromonas sp. SN2]

>gi|333917026|ref|YP_004490758.1| CzcA family heavy metal efflux pump [Delftia sp. Cs1-4]

>gi|334132261|ref|ZP_08506022.1| Cobalt-zinc-cadmium resistance protein CzcA [Methyloversatilis universalis FAM5]

>gi|334141723|ref|YP_004534930.1| heavy metal efflux pump CzcA [Novosphingobium sp. PP1Y]

>gi|334142077|ref|YP_004535284.1| AcrB/AcrD/AcrF family protein [Novosphingobium sp. PP1Y]

>gi|334346550|ref|YP_004556187.1| CzcA family heavy metal efflux pump [Sphingobium chlorophenolicum L-1]

>gi|334836901|gb|EGM15687.1| putative cation efflux system protein [Pseudomonas aeruginosa 152504]

>gi|334837407|gb|EGM16170.1| putative cation efflux system protein [Pseudomonas aeruginosa 138244]

>gi|334843245|gb|EGM21837.1| Resistance-Nodulation-Cell Division (RND) divalent metal cation efflux transporter CzcA [Pseudomonas aeruginosa 152504]

>gi|335042895|ref|ZP_08535922.1| putative silver efflux pump [Methylophaga aminisulfidivorans MP]

>gi|335043820|ref|ZP_08536846.1| putative silver efflux pump [Methylophaga aminisulfidivorans MP]

>gi|335419097|ref|ZP_08550155.1| cobalt/zinc/cadmium efflux RND transporter permease [Salinisphaera shabanensis E1L3A]

>gi|336317030|ref|ZP_08571908.1| heavy metal efflux pump, cobalt-zinc-cadmium [Rheinheimera sp. A13L]

>gi|338175831|ref|YP_004652641.1| cnrA gene product [Parachlamydia acanthamoebae UV-7]

>gi|338176511|ref|YP_004653321.1| nccA gene product [Parachlamydia acanthamoebae UV-7]

>gi|338738362|ref|YP_004675324.1| HME family RND efflux transporter translocase subunit [Hyphomicrobium sp. MC1]

>gi|338781836|gb|EGP46216.1| cation efflux system protein CZCA [Achromobacter xylosoxidans AXX-A]

>gi|338974639|ref|ZP_08629997.1| cobalt-zinc-cadmium resistance protein CzcA [Bradyrhizobiaceae bacterium SG-6C]

>gi|339323643|ref|YP_004682537.1| czcA2 gene product [Cupriavidus necator N-1]

>gi|339329136|ref|YP_004688828.1| cation efflux system protein CzcA [Cupriavidus necator N-1]

>gi|339484483|ref|YP_004696269.1| CzcA family heavy metal efflux pump [Nitrosomonas sp. Is79A3]

>gi|339486916|ref|YP_004701444.1| CzcA family heavy metal efflux protein [Pseudomonas putida S16]

>gi|339490126|ref|YP_004704654.1| CzcA family heavy metal efflux protein [Pseudomonas putida S16]

>gi|339493321|ref|YP_004713614.1| cation efflux family protein [Pseudomonas stutzeri ATCC 17588 = LMG 11199]

>gi|340787707|ref|YP_004753172.1| RND divalent metal cation efflux transporter [Collimonas fungivorans Ter331]

>gi|341614100|ref|ZP_08700969.1| metal ion efflux RND protein family protein [Citromicrobium sp. JLT1363]

>gi|344169402|emb|CCA81749.1| cation efflux system protein, heavy metal resistance [blood disease bacterium R229]

>gi|344172336|emb|CCA84970.1| nickel and cobalt resistance protein cnrA [Ralstonia syzygii R24]

>gi|344175061|emb|CCA87697.1| cation efflux system protein, heavy metal resistance [Ralstonia syzygii R24]

>gi|344924661|ref|ZP_08778122.1| cation efflux system protein czcA [Candidatus Odyssella thessalonicensis L13]

>gi|346726896|ref|YP_004853565.1| silver efflux pump [Xanthomonas axonopodis pv. citrumelo F1]

>gi|347529405|ref|YP_004836153.1| heavy metal efflux system protein [Sphingobium sp. SYK-6]

>gi|347739114|ref|ZP_08870453.1| CzcA family heavy metal efflux protein [Azospirillum amazonense Y2]

>gi|347758962|ref|YP_004866524.1| cobalt-zinc-cadmium resistance protein czcA [Micavibrio aeruginosavorus ARL-13]

>gi|355642413|ref|ZP_09052744.1| cobalt-zinc-cadmium resistance protein czcA [Pseudomonas sp. 2_1_26]

>gi|357416465|ref|YP_004929485.1| putative Cobalt-zinc-cadmium resistance protein [Pseudoxanthomonas spadix BD-a59]

>gi|357417383|ref|YP_004930403.1| putative AcrA/AcrD/AcrF family protein [Pseudoxanthomonas spadix BD-a59]

>gi|357974053|ref|ZP_09138024.1| heavy metal efflux pump CzcA [Sphingomonas sp. KC8]

>gi|357974819|ref|ZP_09138790.1| heavy metal efflux pump CzcA [Sphingomonas sp. KC8]

>gi|357975181|ref|ZP_09139152.1| heavy metal efflux pump, CzcA family protein [Sphingomonas sp. KC8]

>gi|357977106|ref|ZP_09141077.1| heavy metal efflux pump CzcA [Sphingomonas sp. KC8]

>gi|357977121|ref|ZP_09141092.1| heavy metal efflux pump CzcA [Sphingomonas sp. KC8]

>gi|357977178|ref|ZP_09141149.1| heavy metal efflux system protein [Sphingomonas sp. KC8]

>gi|358011007|ref|ZP_09142817.1| putative silver efflux pump [Acinetobacter sp. P8-3-8]

>gi|358013167|ref|ZP_09144977.1| Cation efflux system protein czcA [Acinetobacter sp. P8-3-8]

>gi|358073684|emb|CCE48978.1| Cobalt-zinc-cadmium resistance protein CzcA; Cation efflux system protein CusA [Burkholderia cenocepacia H111]

>gi|358447934|ref|ZP_09158443.1| cobalt/zinc/cadmium efflux RND transporter permease [Marinobacter manganoxydans MnI7-9]

>gi|359399759|ref|ZP_09192754.1| metal ion efflux RND protein family protein [Novosphingobium pentaromativorans US6-1]

>gi|359400403|ref|ZP_09193387.1| Heavy metal efflux pump CzcA [Novosphingobium pentaromativorans US6-1]

>gi|359401590|ref|ZP_09194558.1| AcrB/AcrD/AcrF family protein [Novosphingobium pentaromativorans US6-1]

>gi|359401854|ref|ZP_09194811.1| heavy metal efflux pump CzcA [Novosphingobium pentaromativorans US6-1]

>gi|359401882|ref|ZP_09194838.1| Heavy metal efflux pump CzcA [Novosphingobium pentaromativorans US6-1]

>gi|359401914|ref|ZP_09194869.1| AcrB/AcrD/AcrF family protein [Novosphingobium pentaromativorans US6-1]

>gi|359402453|ref|ZP_09195363.1| AcrB/AcrD/AcrF family protein [Novosphingobium pentaromativorans US6-1]

>gi|359428767|ref|ZP_09219797.1| putative heavy metal efflux system protein [Acinetobacter sp. NBRC 100985]

>gi|359436567|ref|ZP_09226663.1| protein HelA [Pseudoalteromonas sp. BSi20311]

>gi|359436689|ref|ZP_09226780.1| cation efflux system protein CzcA [Pseudoalteromonas sp. BSi20311]

>gi|359437352|ref|ZP_09227420.1| cation efflux system protein CzcA [Pseudoalteromonas sp. BSi20311]

>gi|359438943|ref|ZP_09228925.1| protein HelA [Pseudoalteromonas sp. BSi20311]

>gi|359443786|ref|ZP_09233607.1| cation efflux system protein CzcA [Pseudoalteromonas sp. BSi20429]

>gi|359444257|ref|ZP_09234057.1| protein HelA [Pseudoalteromonas sp. BSi20439]

>gi|359444779|ref|ZP_09234546.1| cation efflux system protein CzcA [Pseudoalteromonas sp. BSi20439]

>gi|359446483|ref|ZP_09236154.1| cation efflux system protein CzcA [Pseudoalteromonas sp. BSi20439]

>gi|359449102|ref|ZP_09238602.1| protein HelA [Pseudoalteromonas sp. BSi20480]

>gi|359453373|ref|ZP_09242692.1| cation efflux system protein CzcA [Pseudoalteromonas sp. BSi20495]

>gi|359783549|ref|ZP_09286762.1| cation efflux family protein [Pseudomonas psychrotolerans L19]

>gi|365893568|ref|ZP_09431740.1| Nickel and cobalt resistance protein cnrA [Bradyrhizobium sp. STM 3843]

>gi|367474195|ref|ZP_09473716.1| Heavy metal efflux pump CzcA [Bradyrhizobium sp. ORS 285]

>gi|371453984|gb|EHN66995.1| cobalt-zinc-cadmium resistance protein,CzcA [Comamonas testosteroni ATCC 11996]

>gi|372266776|ref|ZP_09502824.1| cobalt/zinc/cadmium efflux RND transporter permease [Alteromonas sp. S89]

>gi|372487808|ref|YP_005027373.1| heavy metal efflux pump [Dechlorosoma suillum PS]

>gi|374262809|ref|ZP_09621370.1| HelA protein [Legionella drancourtii LLAP12]

>gi|374575864|ref|ZP_09648960.1| heavy metal efflux pump, cobalt-zinc-cadmium [Bradyrhizobium sp. WSM471]

>gi|374705989|ref|ZP_09712859.1| CzcA family heavy metal efflux pump [Pseudomonas sp. S9]

>gi|375044787|gb|EHS37379.1| Resistance-Nodulation-Cell Division (RND) divalent metal cation efflux transporter CzcA [Pseudomonas aeruginosa MPAO1/P1]

>gi|375111304|ref|ZP_09757515.1| cobalt/zinc/cadmium efflux RND transporter permease [Alishewanella jeotgali KCTC 22429]

>gi|375136297|ref|YP_004996947.1| czcA gene product [Acinetobacter calcoaceticus PHEA-2]

>gi|377811797|ref|YP_005044237.1| czcA gene product [Burkholderia sp. YI23]

>gi|378777981|ref|YP_005186419.1| cobalt/zinc/cadmium efflux RND transporter, permease HelA [Legionella pneumophila subsp. pneumophila ATCC 43290]

>gi|379067099|gb|EHY79842.1| cation efflux family protein, partial [Pseudomonas stutzeri ATCC 14405 = CCUG 16156]

>gi|381153649|ref|ZP_09865518.1| heavy metal efflux pump, cobalt-zinc-cadmium [Methylomicrobium album BG8]

>gi|381172319|ref|ZP_09881450.1| cobalt-zinc-cadmium resistance protein CzcA [Xanthomonas citri pv. mangiferaeindicae LMG 941]

>gi|381196023|ref|ZP_09903365.1| Cation efflux system protein czcA [Acinetobacter lwoffii WJ10621]

>gi|381202056|ref|ZP_09909174.1| CzcA family heavy metal efflux pump [Sphingobium yanoikuyae XLDN2-5]

>gi|381393514|ref|ZP_09919235.1| nickel and cobalt resistance protein CnrA [Glaciecola punicea DSM 14233 = ACAM 611]

>gi|383771497|ref|YP_005450562.1| heavy metal efflux pump CzcA [Bradyrhizobium sp. S23321]

>gi|383936787|ref|ZP_09990207.1| protein helA [Rheinheimera nanhaiensis E407-8]

>gi|383937120|ref|ZP_09990531.1| protein helA [Rheinheimera nanhaiensis E407-8]

>gi|384218438|ref|YP_005609604.1| hypothetical protein [Bradyrhizobium japonicum USDA 6]

>gi|384425915|ref|YP_005635272.1| czcA gene product [Xanthomonas campestris pv. raphani 756C]

>gi|384928980|ref|ZP_10028875.1| CzcA family heavy metal efflux protein [Burkholderia sp. SJ98]

>gi|385205636|ref|ZP_10032506.1| heavy metal efflux pump, cobalt-zinc-cadmium [Burkholderia sp. Ch1-1]

>gi|386019860|ref|YP_005937884.1| acrB gene product [Pseudomonas stutzeri DSM 4166]

>gi|386022119|ref|YP_005940144.1| cation efflux family protein [Pseudomonas stutzeri DSM 4166]

>gi|386058582|ref|YP_005975104.1| czcA gene product [Pseudomonas aeruginosa M18]

>gi|386286743|ref|ZP_10063930.1| CzcA family heavy metal efflux protein [gamma proteobacterium BDW918]

>gi|386287896|ref|ZP_10065064.1| CzcA family heavy metal efflux protein [gamma proteobacterium BDW918]

>gi|386287913|ref|ZP_10065081.1| CzcA family heavy metal efflux protein [gamma proteobacterium BDW918]

>gi|386288272|ref|ZP_10065427.1| cation efflux system protein [gamma proteobacterium BDW918]

>gi|386312522|ref|YP_006008687.1| CzcA family heavy metal efflux pump [Shewanella putrefaciens 200]

>gi|386315589|ref|YP_006011754.1| cobalt-zinc-cadmium cation efflux system permease, CzcA family [Shewanella putrefaciens 200]

>gi|386335342|ref|YP_006031512.1| czcA gene product [Ralstonia solanacearum Po82]

>gi|386402030|ref|ZP_10086808.1| heavy metal efflux pump, cobalt-zinc-cadmium [Bradyrhizobium sp. WSM1253]

>gi|386402540|ref|ZP_10087318.1| heavy metal efflux pump, cobalt-zinc-cadmium [Bradyrhizobium sp. WSM1253]

>gi|386716602|ref|YP_006182928.1| smmQ2 gene product [Stenotrophomonas maltophilia D457]

>gi|386716608|ref|YP_006182934.1| cusA gene product [Stenotrophomonas maltophilia D457]

>gi|386812127|ref|ZP_10099352.1| heavy metal efflux pump [planctomycete KSU-1]

>gi|387126290|ref|YP_006294895.1| Cobalt-zinc-cadmium resistance protein CzcA [Methylophaga sp. JAM1]

>gi|387131482|ref|YP_006294372.1| Cobalt-zinc-cadmium resistance protein CzcA, Cation efflux system protein CusA [Methylophaga sp. JAM7]

>gi|387891913|ref|YP_006322210.1| cobalt/zinc/cadmium resistance protein CzcA [Pseudomonas fluorescens A506]

>gi|387967123|gb|EIK51433.1| cation efflux family protein [Pseudomonas stutzeri TS44]

>gi|387997433|gb|EIK58762.1| cobalt/zinc/cadmium resistance protein CzcA [Pseudomonas fluorescens SS101]

>gi|388256163|ref|ZP_10133344.1| CzcA cation efflux system protein [Cellvibrio sp. BR]

>gi|388259580|ref|ZP_10136752.1| heavy metal efflux pump, CzcA family [Cellvibrio sp. BR]

>gi|388456999|ref|ZP_10139294.1| cobalt/zinc/cadmium efflux RND transporter permease HelA [Fluoribacter dumoffii Tex-KL]

>gi|388469689|ref|ZP_10143898.1| cobalt/zinc/cadmium resistance protein CzcA [Pseudomonas synxantha BG33R]

>gi|389684222|ref|ZP_10175552.1| cobalt/zinc/cadmium resistance protein CzcA, degenerate [Pseudomonas chlororaphis O6]

>gi|389707079|ref|ZP_10186484.1| Cation efflux system protein czcA [Acinetobacter sp. HA]

>gi|389807259|ref|ZP_10204053.1| heavy metal efflux pump [Rhodanobacter thiooxydans LCS2]

>gi|390189856|emb|CCD32090.1| Heavy metal efflux pump, CzcA family [Methylocystis sp. SC2]

>gi|390929356|gb|EIP86759.1| heavy metal efflux pump CzcA [Burkholderia thailandensis MSMB43]

>gi|390992906|ref|ZP_10263116.1| cobalt-zinc-cadmium resistance protein CzcA [Xanthomonas axonopodis pv. punicae str. LMG 859]

>gi|3914126|sp|Q44586.1|NCCA_ALCXX RecName: Full=Nickel-cobalt-cadmium resistance protein NccA

>gi|392409057|ref|YP_006445664.1| heavy metal efflux pump, cobalt-zinc-cadmium [Desulfomonile tiedjei DSM 6799]

>gi|392423356|ref|YP_006459960.1| RND divalent metal cation efflux transporter [Pseudomonas stutzeri CCUG 29243]

>gi|392535153|ref|ZP_10282290.1| cobalt-zinc-cadmium resistance protein czcA [Pseudoalteromonas arctica A 37-1-2]

>gi|392536754|ref|ZP_10283891.1| cobalt-zinc-cadmium resistance protein czcA [Pseudoalteromonas marina mano4]

>gi|392539224|ref|ZP_10286361.1| heavy metal efflux pump CzcA [Pseudoalteromonas marina mano4]

>gi|392549683|ref|ZP_10296820.1| heavy metal efflux pump CzcA [Pseudoalteromonas spongiae UST010723-006]

>gi|392553829|ref|ZP_10300966.1| cobalt-zinc-cadmium resistance protein CzcA [Pseudoalteromonas undina NCIMB 2128]

>gi|392951664|ref|ZP_10317219.1| cation efflux system protein CzcA [Hydrocarboniphaga effusa AP103]

>gi|392954086|ref|ZP_10319638.1| cation efflux system protein [Hydrocarboniphaga effusa AP103]

>gi|392954520|ref|ZP_10320071.1| AcrB/AcrD/AcrF family protein [Hydrocarboniphaga effusa AP103]

>gi|392954557|ref|ZP_10320108.1| cation efflux system protein CzcA [Hydrocarboniphaga effusa AP103]

>gi|393720104|ref|ZP_10340031.1| heavy metal efflux pump CzcA [Sphingomonas echinoides ATCC 14820]

>gi|393720118|ref|ZP_10340045.1| heavy metal efflux pump CzcA [Sphingomonas echinoides ATCC 14820]

>gi|393762431|ref|ZP_10351058.1| cobalt/zinc/cadmium efflux RND transporter permease [Alishewanella agri BL06]

>gi|393764081|ref|ZP_10352693.1| CzcA family heavy metal efflux protein [Alishewanella agri BL06]

>gi|393764553|ref|ZP_10353158.1| CzcA family heavy metal efflux pump [Methylobacterium sp. GXF4]

>gi|393767674|ref|ZP_10356220.1| cobalt-zinc-cadmium resistance protein czcA [Methylobacterium sp. GXF4]

>gi|393768478|ref|ZP_10357016.1| CzcA family heavy metal efflux protein [Methylobacterium sp. GXF4]

>gi|393768563|ref|ZP_10357100.1| RND divalent metal cation efflux transporter CzcA [Methylobacterium sp. GXF4]

>gi|393770760|ref|ZP_10359238.1| Nickel-cobalt-cadmium resistance protein NccA [Novosphingobium sp. Rr 2-17]

>gi|393774726|ref|ZP_10363081.1| metal ion efflux RND protein family protein [Novosphingobium sp. Rr 2-17]

>gi|393777610|ref|ZP_10365901.1| heavy metal efflux pump, czca family [Ralstonia sp. PBA]

>gi|395444190|ref|YP_006384443.1| CzcA family heavy metal efflux protein [Pseudomonas putida ND6]

>gi|395494243|ref|ZP_10425822.1| heavy metal efflux pump CzcA [Sphingomonas sp. PAMC 26617]

>gi|395500740|ref|ZP_10432319.1| cobalt/zinc/cadmium resistance protein CzcA [Pseudomonas sp. PAMC 25886]

>gi|395523726|gb|EJG11815.1| cation efflux system protein CzcA [Acinetobacter baumannii OIFC137]

>gi|395557530|gb|EJG23531.1| cation efflux system protein CzcA [Acinetobacter baumannii OIFC143]

>gi|395651460|ref|ZP_10439310.1| cobalt/zinc/cadmium resistance protein CzcA [Pseudomonas extremaustralis 14-3 substr. 14-3b]

>gi|395760841|ref|ZP_10441510.1| RND divalent metal cation efflux transporter [Janthinobacterium sp. PAMC 25724]

>gi|395798422|ref|ZP_10477707.1| cobalt/zinc/cadmium resistance protein CzcA [Pseudomonas sp. Ag1]

>gi|397665139|ref|YP_006506677.1| HelA protein [Legionella pneumophila subsp. pneumophila]

>gi|397666725|ref|YP_006508262.1| HelA protein [Legionella pneumophila subsp. pneumophila]

>gi|397667264|ref|YP_006508801.1| HelA protein [Legionella pneumophila subsp. pneumophila]

>gi|397883485|gb|EJK99971.1| cobalt/zinc/cadmium resistance protein CzcA [Pseudomonas chlororaphis subsp. aureofaciens 30-84]

>gi|398383066|ref|ZP_10541142.1| heavy metal efflux pump, cobalt-zinc-cadmium [Sphingobium sp. AP49]

>gi|398810111|ref|ZP_10568941.1| heavy metal efflux pump, cobalt-zinc-cadmium [Variovorax sp. CF313]

>gi|398821619|ref|ZP_10580060.1| heavy metal efflux pump, cobalt-zinc-cadmium [Bradyrhizobium sp. YR681]

>gi|398837248|ref|ZP_10594557.1| heavy metal efflux pump, cobalt-zinc-cadmium [Herbaspirillum sp. YR522]

>gi|398845356|ref|ZP_10602394.1| heavy metal efflux pump, cobalt-zinc-cadmium [Pseudomonas sp. GM84]

>gi|398853065|ref|ZP_10609697.1| heavy metal efflux pump, cobalt-zinc-cadmium [Pseudomonas sp. GM80]

>gi|398906765|ref|ZP_10653607.1| heavy metal efflux pump, cobalt-zinc-cadmium [Pseudomonas sp. GM50]

>gi|398970358|ref|ZP_10683280.1| heavy metal efflux pump, cobalt-zinc-cadmium [Pseudomonas sp. GM30]

>gi|398974085|ref|ZP_10684822.1| heavy metal efflux pump, cobalt-zinc-cadmium [Pseudomonas sp. GM25]

>gi|398990328|ref|ZP_10693519.1| heavy metal efflux pump, cobalt-zinc-cadmium [Pseudomonas sp. GM24]

>gi|399003422|ref|ZP_10706086.1| heavy metal efflux pump, cobalt-zinc-cadmium [Pseudomonas sp. GM18]

>gi|399009884|ref|ZP_10712288.1| heavy metal efflux pump, cobalt-zinc-cadmium [Pseudomonas sp. GM17]

>gi|399017793|ref|ZP_10719982.1| heavy metal efflux pump, cobalt-zinc-cadmium [Herbaspirillum sp. CF444]

>gi|399061950|ref|ZP_10746381.1| heavy metal efflux pump, cobalt-zinc-cadmium [Novosphingobium sp. AP12]

>gi|399064329|ref|ZP_10747309.1| heavy metal efflux pump, cobalt-zinc-cadmium [Novosphingobium sp. AP12]

>gi|399074742|ref|ZP_10751177.1| heavy metal efflux pump, cobalt-zinc-cadmium [Caulobacter sp. AP07]

>gi|39935125|ref|NP_947401.1| czcA2 gene product [Rhodopseudomonas palustris CGA009]

>gi|420932|pir||G47056 nickel-cobalt resistance determinant structural protein CnrA - Alcaligenes eutrophus

>gi|46445922|ref|YP_007287.1| czcA gene product [Candidatus Protochlamydia amoebophila UWE25]

>gi|50086352|ref|YP_047862.1| RND divalent metal cation efflux transporter [Acinetobacter sp. ADP1]

>gi|52841242|ref|YP_095041.1| cobalt/zinc/cadmium efflux RND transporter permease HelA [Legionella pneumophila subsp. pneumophila str. Philadelphia 1]

>gi|53716916|ref|YP_105698.1| czcA gene product [Burkholderia mallei ATCC 23344]

>gi|53722062|ref|YP_111047.1| czcA gene product [Burkholderia pseudomallei K96243]

>gi|54293987|ref|YP_126402.1| HelA protein [Legionella pneumophila str. Lens]

>gi|54298312|ref|YP_124681.1| HelA protein [Legionella pneumophila str. Paris]

>gi|56130692|ref|YP_145595.1| cation efflux system transmembrane protein [Ralstonia metallidurans CH34]

>gi|56130753|ref|YP_145656.1| putative nickel and cobalt resistance protein [Ralstonia metallidurans CH34]

>gi|56459886|ref|YP_155167.1| Co/Zn/Cd efflux system membrane protein [Idiomarina loihiensis L2TR]

>gi|56460733|ref|YP_156014.1| metal efflux system membrane protein [Idiomarina loihiensis L2TR]

>gi|56550639|ref|YP_161705.1| nickel and cobalt resistance protein cnrA [Cupriavidus metallidurans CH34]

>gi|5921518|emb|CAB56471.1| czrA protein [Pseudomonas aeruginosa]

>gi|66048027|ref|YP_237868.1| heavy metal efflux pump CzcA [Pseudomonas syringae pv. syringae B728a]

>gi|70732532|ref|YP_262295.1| czcA gene product [Pseudomonas protegens Pf-5]

>gi|71280615|ref|YP_268689.1| RND efflux system protein [Colwellia psychrerythraea 34H]

>gi|71737734|ref|YP_276927.1| cation efflux family protein [Pseudomonas syringae pv. phaseolicola 1448A]

>gi|73537801|ref|YP_298168.1| heavy metal efflux pump CzcA [Ralstonia eutropha JMP134]

>gi|74317345|ref|YP_315085.1| heavy metal efflux pump CzcA [Thiobacillus denitrificans ATCC 25259]

>gi|75675649|ref|YP_318070.1| heavy metal efflux pump CzcA [Nitrobacter winogradskyi Nb-255]

>gi|77164840|ref|YP_343365.1| heavy metal efflux pump [Nitrosococcus oceani ATCC 19707]

>gi|77164912|ref|YP_343437.1| heavy metal efflux pump [Nitrosococcus oceani ATCC 19707]

>gi|77359534|ref|YP_339109.1| cobalt-zinc-cadmium resistance protein czcA [Pseudoalteromonas haloplanktis TAC125]

>gi|77459843|ref|YP_349350.1| heavy metal efflux pump CzcA [Pseudomonas fluorescens Pf0-1]

>gi|78049815|ref|YP_365990.1| RND superfamily protein [Xanthomonas campestris pv. vesicatoria str. 85-10]

>gi|78063278|ref|YP_373186.1| CzcA family heavy metal efflux protein [Burkholderia sp. 383]

>gi|78486433|ref|YP_392358.1| heavy metal efflux pump CzcA [Thiomicrospira crunogena XCL-2]

>gi|82703227|ref|YP_412793.1| heavy metal efflux pump CzcA [Nitrosospira multiformis ATCC 25196]

>gi|83718363|ref|YP_439571.1| heavy metal efflux pump CzcA [Burkholderia thailandensis E264]

>gi|83748417|ref|ZP_00945440.1| Cobalt-zinc-cadmium resistance protein (czcA) [Ralstonia solanacearum UW551]

>gi|85373714|ref|YP_457776.1| metal ion efflux RND protein family protein [Erythrobacter litoralis HTCC2594]

>gi|85708271|ref|ZP_01039337.1| metal ion efflux RND protein family protein [Erythrobacter sp. NAP1]

>gi|85711399|ref|ZP_01042458.1| Metal efflux system membrane component [Idiomarina baltica OS145]

>gi|85715525|ref|ZP_01046506.1| heavy metal efflux pump CzcA [Nitrobacter sp. Nb-311A]

>gi|85717496|ref|ZP_01048443.1| metal ion efflux RND protein family protein [Nitrobacter sp. Nb-311A]

>gi|87200162|ref|YP_497419.1| heavy metal efflux pump CzcA [Novosphingobium aromaticivorans DSM 12444]

>gi|88707079|ref|ZP_01104774.1| Cation efflux system protein czcA [Congregibacter litoralis KT71]

>gi|88858182|ref|ZP_01132824.1| Cobalt-zinc-cadmium resistance protein czcA [Pseudoalteromonas tunicata D2]

>gi|90021600|ref|YP_527427.1| cation efflux system protein [Saccharophagus degradans 2-40]

>gi|90023408|ref|YP_529235.1| Co/Zn/Cd efflux system membrane protein [Saccharophagus degradans 2-40]

>gi|91776901|ref|YP_546657.1| heavy metal efflux pump CzcA [Methylobacillus flagellatus KT]

>gi|91776916|ref|YP_546672.1| heavy metal efflux pump CzcA [Methylobacillus flagellatus KT]

>gi|91779388|ref|YP_554596.1| RND heavy metal efflux pump, CzcA subunit [Burkholderia xenovorans LB400]

>gi|91976621|ref|YP_569280.1| heavy metal efflux pump CzcA [Rhodopseudomonas palustris BisB5]

>gi|91977807|ref|YP_570466.1| heavy metal efflux pump CzcA [Rhodopseudomonas palustris BisB5]

>gi|91978111|ref|YP_570770.1| heavy metal efflux pump CzcA [Rhodopseudomonas palustris BisB5]

>gi|92109657|ref|YP_571944.1| heavy metal efflux pump CzcA [Nitrobacter hamburgensis X14]

>gi|92117395|ref|YP_577124.1| heavy metal efflux pump CzcA [Nitrobacter hamburgensis X14]

>gi|92117430|ref|YP_577159.1| heavy metal efflux pump CzcA [Nitrobacter hamburgensis X14]

>gi|94313393|ref|YP_586602.1| heavy metal cation tricomponent efflux pump HmuA [Cupriavidus metallidurans CH34]

>gi|94495714|ref|ZP_01302294.1| metal ion efflux RND protein family protein [Sphingomonas sp. SKA58]

>gi|94498302|ref|ZP_01304861.1| metal ion efflux RND protein family protein [Sphingomonas sp. SKA58]
