# Supplementary material for: Two RND proteins involved in heavy metal efflux in Caulobacter crescentus belong to separate clusters within proteobacteria
Source: BMC Microbiol. 2013 Apr 11;13:79. doi: 10.1186/1471-2180-13-79 (PMC3637150; doi:10.1186/1471-2180-13-79)
Supplement: Additional file 2: Figure S1 — Sequence conservation profile within the CzrA and NczA orthologous groups. [file 1471-2180-13-79-S2.pdf]

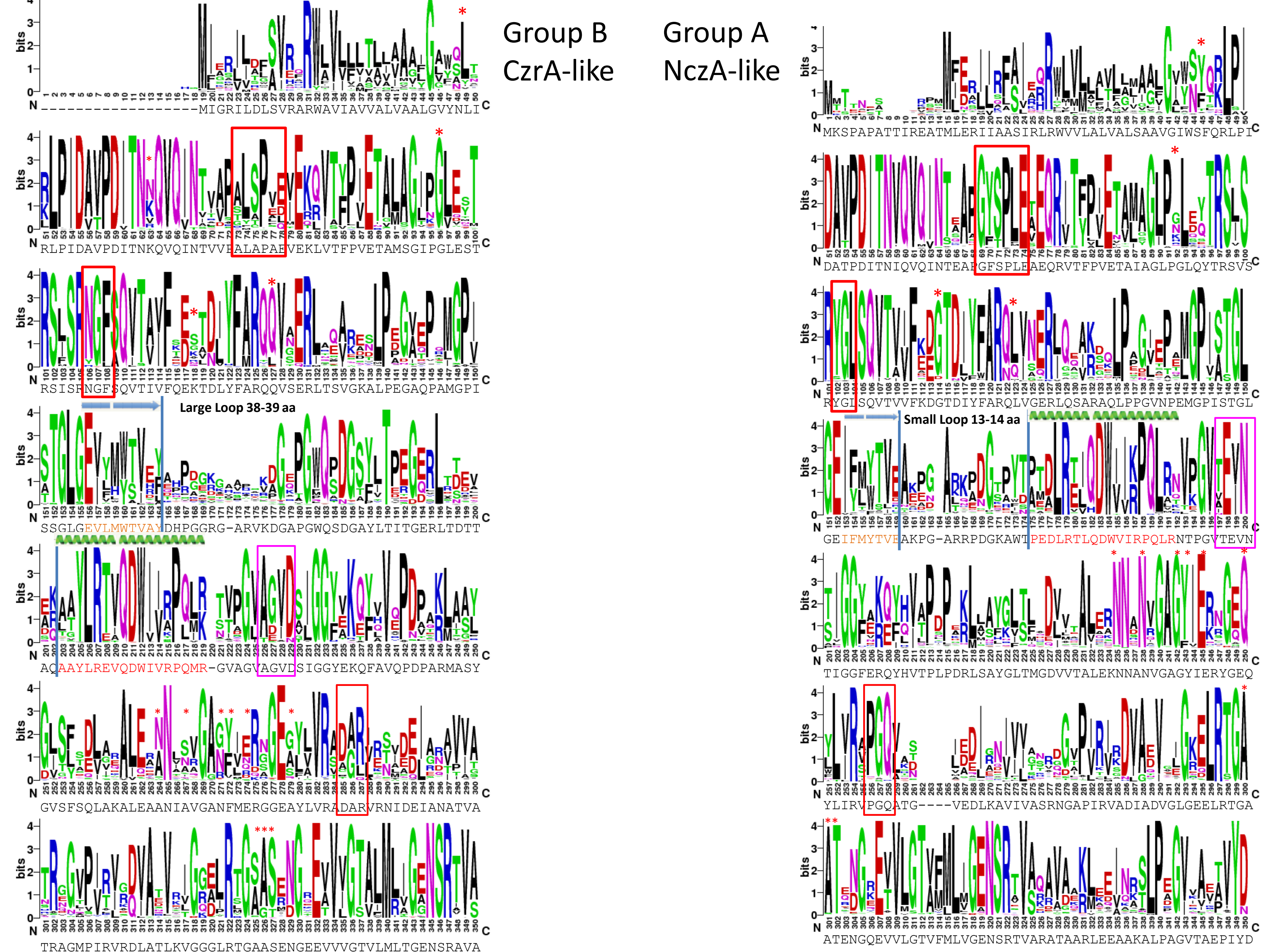
Group B
CzrA-like
Group A
NczA-like
Large Loop 38-39 aa
Small Loop 13-14 aa
-----------------MIGRILDLSVRARWAVIAVVALVAALGVYNLI
RLPIDAVPDITNKQVQINTVVPALAPAEVEKLVTFPVETAMSGIPGLEST
RSISPNGFSQVTIVFQEKTDLYFARQQVSERLTSVGKALPEGAQPAMGPI
SSGLGEVLMWTVAYDHPGGRG-ARVKDGAPGWQSDGAYLTITGERLTDTT
AQAAYLREVQDWIVRPQMR-GVAGVAGVDSIGGYEKQFAVQPDPARMASY
GVSFSQLAKALEAANIAVGANFMERGGEAYLVRADARVRNIDEIANATVA
TRAGMPIRVRDLATLKVGGGLRTGAASENGEEVVVGTVLMLTGENSRAVA
MKSPAPATTIREATMLERIIAASIRLRWVVLALVALSAAVGIWSFQRLPI
DATPDITNIQVQINTEAPGFSPLEAEQRVTFPVETAIAGLPGLQYTRSVS
RYGISQVTVVFKDGTDIYFARQLVGERLQSARAQLPPGVNPEMGPISTGL
GEIFMYTVEAKPG-ARRPDGKAWTPEDLRTLQDWVIRPQLRNTPGVTEVN
TIGGFERQYHVTPLPDRLSAYGLTMGDVVTALEKNNANVGAGYIERYGEQ
YLIRVPGQATG----VEDLKAVIVASRNGAPIRVADIADVGLGEELRTGA
ATENGQEVVLGTVFMLVGENSRTVARATAARLEEAAKALPAGVTAEPIYD

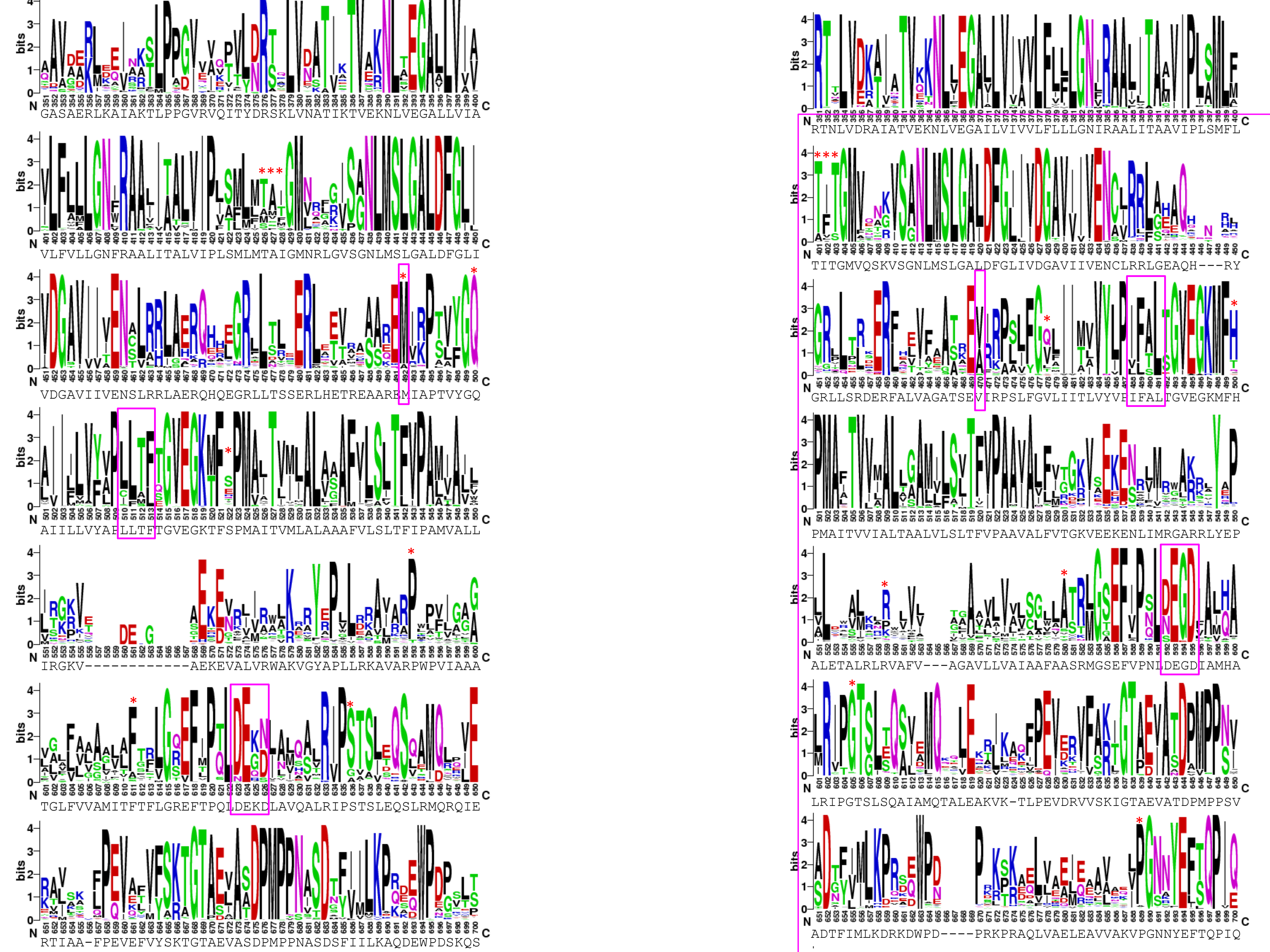
GASAERLKAIAKTLPPGVRVQITYDRSKLVNATIKTVEKNLVEGALLVIA
VLFVLLGNFRAALITALVIPLSMLMTAIGMNRLGVSGNLMSLGALDFGLI
VDGAVIIVENSLRRLAERQHQEGRLLTSSERLHETREAARRMIAPTVYGQ
AIILLVYAPLLTFTGVEGKTFSPMAITVMLALAAAFVLSLTFIPAMVALL
IRGKV-----------AEKEVALVRWAKVGYAPLLRKAVARPWPVIAAA
TGLFVVAMITFTFLGREFTPQLDEKDLAVQALRIPSTSLEQSLRMQRQIE
RTIAA-FPEVEFVYSKTGTAEVASDPMPPNASDSFIILKAQDEWPDSKQS
RTNLVDRAIATVEKNLVEGAILVIVVLFLLLGNIRAALITAAVIPLSMFL
TITGMVQSKVSGNLMSLGALDFGLIVDGAVIIVENCLRRLGEAQH---RY
GRLLSRDERFALVAGATSEVIRPSLFGVLIITLVYVPIFALTGVEGKMFH
PMAITVVIALTAALVLSLTFVPAAVALFVTGKVEEKENLIMRGARRLYEP
ALETALRLRVAFV---AGAVLLVAIAAFAASRMGSEFVPNLDEGDIAMHA
LRIPGTSLSQAIAMQTALEAKVK-TLPEVDRVVSKIGTAEVATDPMPPSV
ADTFIMLKDRKDWPD----PRKPRAQLVAELEAVVAKVPGNNYEFTQPIQ

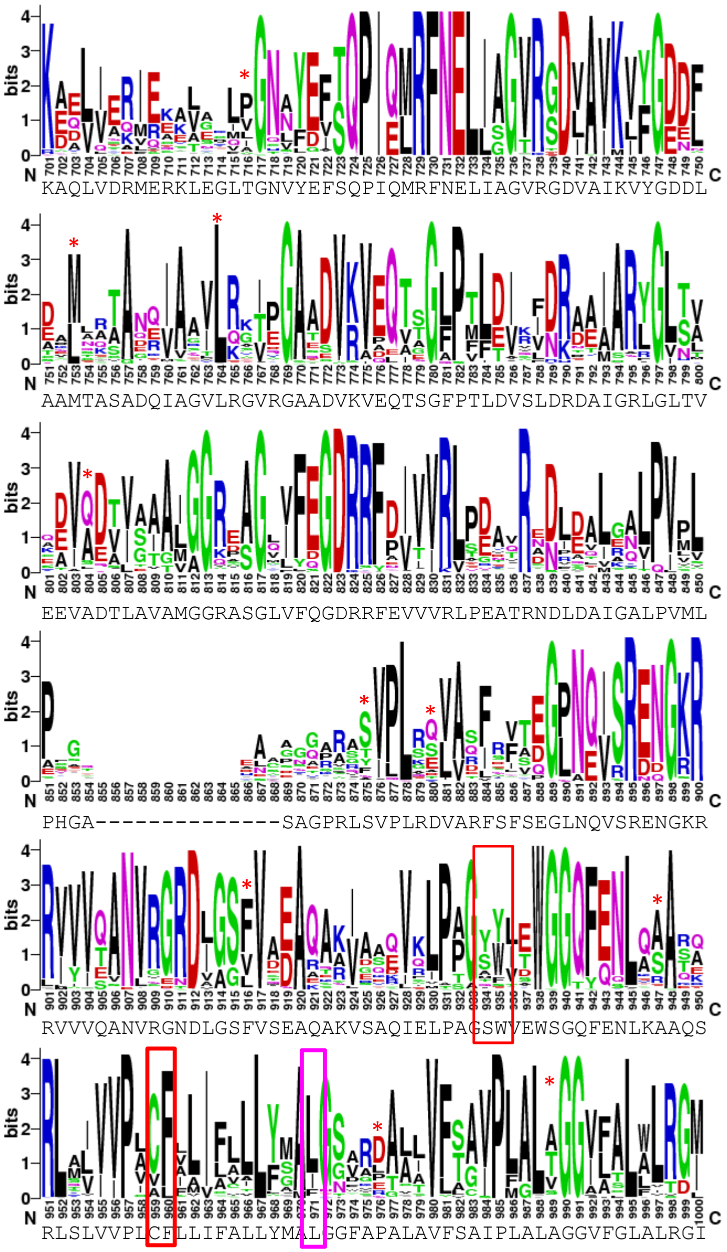
KAQLVDRMERKLEGLTGNVYEFSQPIQMRFNELIAGVRGDVAIKVYGDDL
AAMTASADQIAGVLRGVRGAADVKVEQTSGFPTLDVSLDRDAIGRLGLTV
EEVADTLAVAMGGRASGLVFQGDRRFEVVVRLPEATRNDLDAIGALPVML
PHGA--------------SAGPRLSVPLRDVARFSFSEGLNQVSRENGKR
RVVVQANVRGNDLGSFVSEAQAKVSAQIELPAGSWVEWSGQFENLKAAQS
RLSLVVPLCFLLIFALLYMALGGFAPALAVFSAIPLALAGGVFGLALRGI

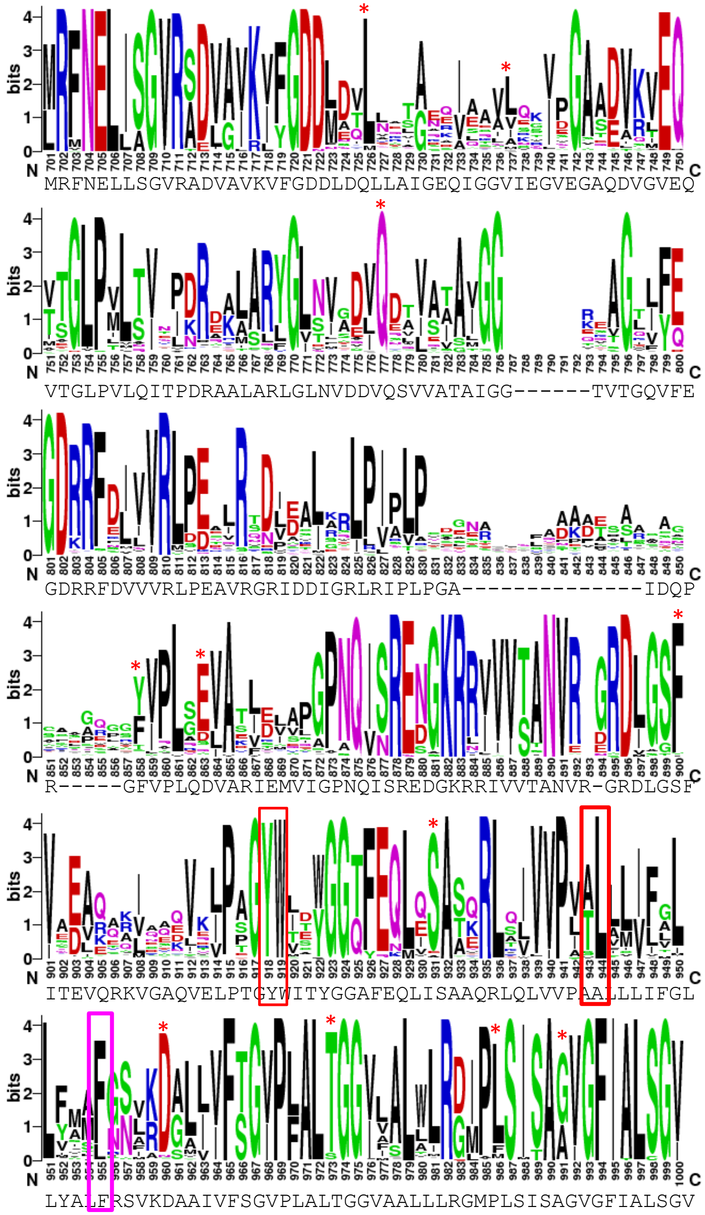
MRFNELLSGVRADVAVKVFGDDLDQLLAIGEQIGGVIEGVEGAQDVGVEQ
VTGLPVLQITPDRAALARLGLNVDDVQSVVATAIGG------TVTGQVFE
GDRRFDVVVVRLPEAVRGRIDDIGRLRIPLPGA-------------IDQP
R-----GFVPLQDVARIEMVIGPNQISREDGKRRIVVTANVR-GRDLGSF
ITEVQRKVGAQVELPTGYWITYGGAFEQLISAAQRLQLVVPAALLLIFGL
LYALFRSVKDAAIVFSGVPLALTGGVAALLLRGMPLSISAGVGFIALSGV

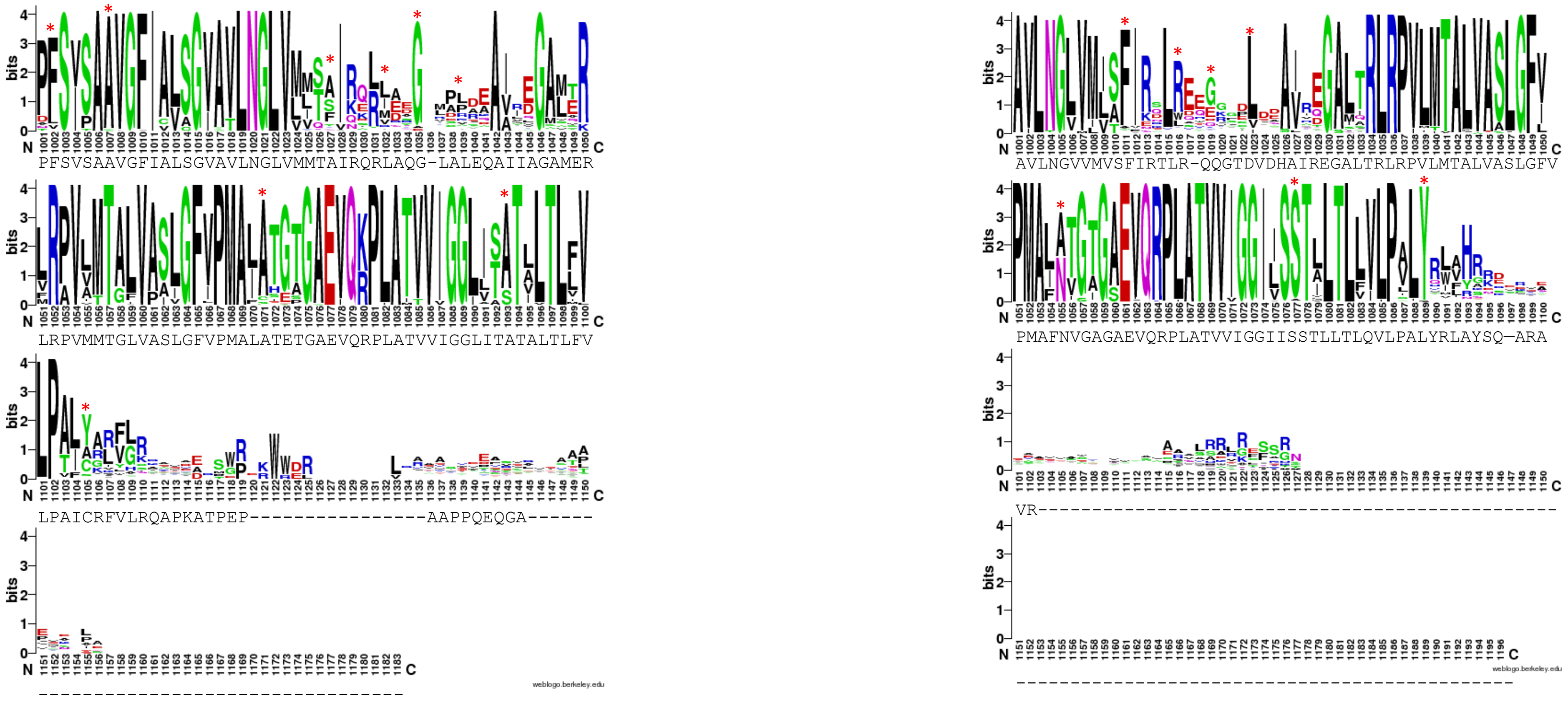

Valencia E. Y., Braz, V. S., Guzzo, C. and Marques, M. V. Two RND proteins involved in heavy metal efflux in *Caulobacter crescentus* belong to separate clusters within Proteobacteria

Supplementary Figure S1: Sequence conservation profile within the CzrA and NczA orthologous groups. Main differences in the sequence conservation profile between the CzrA and NczA orthologous groups (left and right of the image, respectively) are indicated by boxes and asterisks. The motifs are shown in red boxes. The figure was generated using the WebLogo server (http://weblogo.berkeley.edu/).
