# Supplementary material for: Two RND proteins involved in heavy metal efflux in Caulobacter crescentus belong to separate clusters within proteobacteria
Source: BMC Microbiol. 2013 Apr 11;13:79. doi: 10.1186/1471-2180-13-79 (PMC3637150; doi:10.1186/1471-2180-13-79)
Supplement: Additional file 3: Figure S2 — Potential methionine pairs/clusters in CzrA model structure. [file 1471-2180-13-79-S3.pdf]

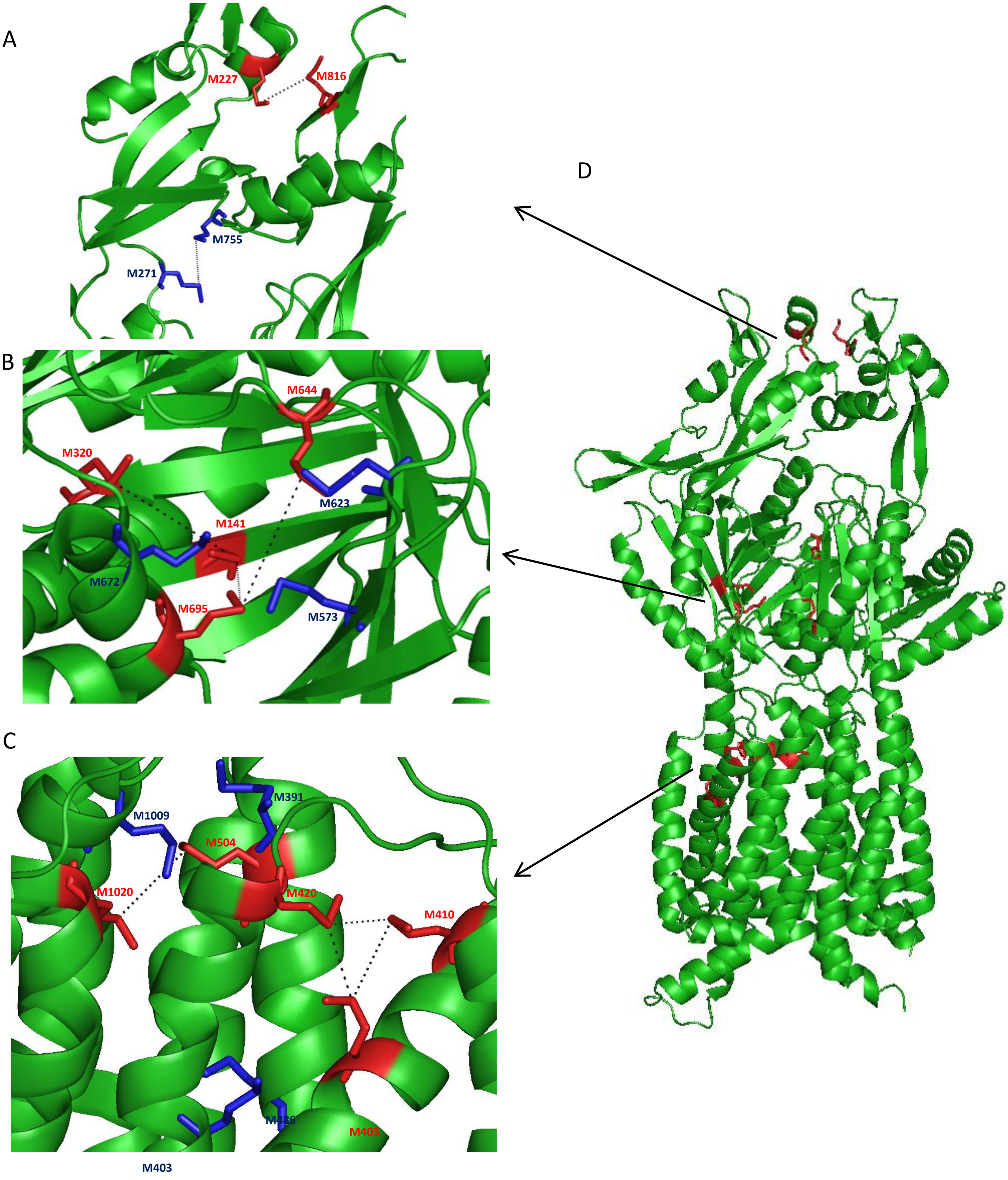

Valencia E. Y., Braz, V. S., Guzzo, C. and Marques, M. V. Two RND proteins involved in heavy metal efflux in *Caulobacter crescentus* belong to separate clusters within Proteobacteria

Supplementary Figure S2: Potential methionine pairs/clusters in CzrA model structure. The structure of CzrA model is shown as a cartoon colored in green. Methionines belonging to CzrA are shown in red sticks and the methionines belonging to CusA (PDBIS: 3K07) are shown in blue sticks. M755-M271 from CusA could correlate to CzrA M227 and M816 (A) and the three essential methionines from CusA, M672, M573, M623 could correlate to two methionines pairs, M695-M644 and M320-M141 (B). Two transmembrane methionine pairs from CusA, M1009-M391 and M403-M486, could correlate to M1020-M391 and the methionine cluster, M420, M410 and M403, respectively (C). All of these potential methionine pairs in CzrA do not build a clear channel in the structure model (D). The structure was generated using PyMOL [http://www.pymol.org.].
